# Optimal Annual COVID-19 Vaccine Boosting Dates Following Previous Booster Vaccination or Breakthrough Infection

**DOI:** 10.1093/cid/ciae559

**Published:** 2024-11-26

**Authors:** Jeffrey P Townsend, Hayley B Hassler, Alex Dornburg

**Affiliations:** Department of Biostatistics, Yale School of Public Health, New Haven, Connecticut, USA; Department of Ecology and Evolutionary Biology, Yale University, New Haven, Connecticut, USA; Program in Computational Biology and Bioinformatics, Yale University, New Haven, Connecticut, USA; Program in Microbiology, Yale University, New Haven, Connecticut, USA; Department of Biostatistics, Yale School of Public Health, New Haven, Connecticut, USA; Interdisciplinary Graduate Program in Quantitative Biosciences, Georgia Institute of Technology, Atlanta, Georgia, USA; Department of Bioinformatics and Genomics, University of North Carolina at Charlotte, Charlotte, North Carolina, USA

**Keywords:** SARS-CoV-2, vaccine, booster, COVID-19, seasonality

## Abstract

**Background:**

COVID-19 booster vaccinations mitigate transmission and reduce the morbidity and mortality associated with infection. However, the optimal date for booster administration remains uncertain. Geographic variation in infection rates throughout the year makes it challenging to intuit the best yearly booster administration date to effectively prevent infection, and also challenging to provide best guidance on how to alter booster administration in response to a breakthrough infection.

**Methods:**

We leveraged longitudinal antibody and reinfection probabilities with spatiotemporal projections of COVID-19 incidence to develop a geographically informed approach to optimizing the timing of booster vaccination. We assessed the delay in booster vaccination that is warranted following breakthrough infections whenever they occur during the year, enabling a personalized assessment of optimal timing that acknowledges and respects diversity of COVID-19 immune status, addressing a substantial barrier to uptake.

**Results:**

Yearly booster vaccination on any date is beneficial to prevention of infection. However, each location exhibits as much as a 3–4-fold range in degree of protection by date of uptake. Optimal COVID-19 booster vaccination dates are location-specific, typically in early autumn in the Northern Hemisphere. Infection late in the interval between boosts substantially alters the optimal boosting date.

**Conclusions:**

Considerable benefit accrues from aptly timing COVID-19 booster vaccination campaigns, which can be tailored to specific locations. Individuals can acquire the greatest benefit from booster vaccination by timing it optimally, including delaying in cases of infection late in the interval between boosts. These results provide location-specific guidance for public health policy, healthcare provider recommendations, and individual decision-making.

## Teaser

Optimal timing for COVID-19 boosters improves their efficacy. There is an optimal delay of boosters after breakthrough infection. Our analysis enables beneficial personalization of booster schedules on the basis of infection history. This personalization improves public health.

Vaccines against coronavirus disease 2019 (COVID-19) confer protection against infection [1] and severe disease [2]. In New York City alone, vaccination prevented nearly 300 000 cases, 50 000 hospitalizations, and 8500 deaths in 7 key months [3]. For long-term protection, booster vaccination is necessary to address the waning of severe acute respiratory syndrome coronavirus 2 (SARS-CoV-2) antibody levels [4]. However, the date when it is optimal to administer yearly booster vaccinations is not clear. It is complicated by the substantial spatiotemporal variation in incidence exhibited by SARS-CoV-2 [5]. Aligning booster timing to provide peak protection during peak incidence could be crucial to maximizing benefits.

The ascertainment of optimal dates for yearly booster vaccination is important not only for those who have not recently been infected, but also for those who experience breakthrough infections. The production of antibodies for recent breakthrough infection would offset benefits of an immediate booster vaccination [6, 7]. Current guidance suggests delay of reinforcement of immunity to later in the year, with the US Centers for Disease Control and Prevention (CDC) currently recommending a delay of up to 3 months [8]. However, this guidance has not been rigorously evaluated, creating a significant barrier to vaccine uptake. Therefore, it is vital to establish policies assessing the optimal timing of boosters and acknowledging the impact of breakthrough infections.

The question of when to boost has been extensively researched for influenza, which exhibits seasonal infection surges associated with climate [9] and for which analyses of extensive endemic seasonal incidence data have been collected and analyzed [10]. Consequently, the US CDC recommends influenza boosters between September and October in the United States [11]. In countries that span temperate and tropical latitudes such as China, the optimal timing of yearly influenza boosters varies within the nation by as much as several months [12]. These findings regarding influenza suggest performing similar seasonality analyses of 2019–2024 COVID-19 infection data. However, there are far fewer years of COVID-19 seasonal infection prevalence data on which to base predictions. Moreover, COVID-19 infections during recent years do not reflect endemic seasonality, and instead have been characterized by pandemic surges driven by historical contingencies [13], early saltations of viral evolution [14], and wholly naive versus differentially exposed immunological states of populations [15]. Therefore, an alternate approach to evaluating optimal timing is necessary until there is sufficient accumulation of long-term empirical data on seasonality.

Such an alternate approach has been enabled by recent research based on ample coronavirus incidence data that documented substantial spatiotemporal heterogeneity in peak incidence across the Northern Hemisphere [16]. These results largely match typical respiratory virus incidence patterns, featuring peaks of “flu and cold season” with some specificity in each locality. However, in isolation, these results give no direct guidance regarding when to administer booster vaccinations. The optimal timing of booster vaccinations depends jointly on the risk of infection due to seasonality and on the long-term waning of protection subsequent to booster vaccination [17]. Rigorous estimates of long-term protection subsequent to booster vaccination have been obtained by leveraging longitudinal antibody and reinfection data from the close evolutionary relatives of SARS-CoV-2 as well as infection and antibody data on SARS-CoV-2 to estimate the durability of immunity following natural infection [18], primary vaccination [19], and boosting [20, 21]. These analyses, subsequently validated by comparison to empirical data [22–24], indicate that statistical approaches derived from evolutionary medicine can illuminate reinfection risks with high accuracy and precision. Yet, the impact on yearly infection of administering yearly booster vaccinations at specific times in specific locations remains unknown—as does the impact of breakthrough infections at specific times of the year on the optimal timing of booster vaccination.

Here we integrated the waxing probabilities of infection subsequent to antigen exposure with projections of the expected seasonal variation in frequency of infection for endemic SARS-CoV-2 [16] to perform a high-resolution investigation of prospective timings of booster vaccinations that maximally curtail infection. We evaluated the optimal boosting date over the year for individuals who have not been infected over the previous year. Then we analyzed similar optimal timings for individuals who have been infected during that year to determine any advantage to delaying the booster, depending on the date of infection. Such knowledge of optimal booster vaccination timing will be helpful for physician and individual decision-making in the context of ongoing endemic disease, and is crucial for effective vaccination policy that suppresses morbidity and mortality as a consequence of COVID-19.

## METHODS

We quantified relative monthly probabilities of infection based on seasonal incidence predictions for endemic COVID-19 [16]. These incidences were inferred using a phylogenetic ancestral and descendant state approach leveraging long-term data on the incidence of circulating human coronaviruses in twelve locations within the Northern Hemisphere: Rochester, MN, United States; New York City, NY, United States; Edinburgh, United Kingdom; Stockholm, Sweden; Trøndelag, Norway; Gothenburg, Sweden; Amsterdam, the Netherlands; South Korea (nationwide); Yamagata, Japan; Guangzhou, China; Sarlahi, Nepal; and Beersheba, Israel [16]. These projections were robust to the choice of the model of trait evolution as well as the choice of molecular trees, relative phylogenetic chronograms, and non-recombinant alignments.

These seasonal incidence predictions are derived from long-term endemic coronavirus prevalences that were not impacted by vaccinations or other interventions. However, an individual recently vaccinated against SARS-CoV-2 will experience a lower risk of infection than an unvaccinated individual in an endemic scenario with no interventions. Therefore, over the first month following each date of booster vaccination, we reduced the seasonality-based probability of infection by the daily proportional protection demonstrated in Pfizer-BioNtech booster (BNT162b2) vaccination following 2 primary vaccinations [25]. This vaccine-based daily probability of infection was calculated via linear interpolation between the probability of no breakthrough infection at clinical trial sampling times post–booster vaccination (1 week and 2 weeks), at peak vaccination at 1 month, and at 1 year post-vaccination. For the second to twelfth months following the booster date, reductions in the seasonal probability of infection due to booster vaccination were quantified by analysis of antibody waning and corresponding infection probability [21].

Infection probabilities were associated with antibody levels based on empirical data supplemented by an ancestral and descendent states analysis of long-term antibody data on healthy individuals who experienced other human-infecting coronaviruses [18, 26]. This analysis used a maximum-likelihood molecular phylogeny of human-infecting coronaviruses and their peak-normalized optical density levels of blood-based immunoglobulin G (IgG) antibodies to nucleocapsid protein, spike protein, and whole-virus lysate over time, coupled with reinfection data. The resulting probabilities of reinfection provided probabilistic times to reinfection after recovery under conditions of endemic transmission for SARS-CoV-2. The antibody waning profiles and infection probabilities from this approach have proven consistent with multiple time points reported in subsequent, but shorter-term, wholly empirical studies [22–24].

For each day of the year, we calculated the cumulative yearly probability of infection, wherein the probabilities of infection each day were computed as the probability of not being infected all previous days multiplied by the probability of being infected on the day of interest. The booster vaccination date on which the cumulative yearly probability of infection is at its lowest represents the optimal yearly booster vaccination date.

To examine optimal delays of boosting in the case of a breakthrough SARS-CoV-2 infection, we modified our approach above to address breakthrough infections occurring on each date by renewing protection at the point of breakthrough infection. During the first month after breakthrough infection, reductions in the probabilities of breakthrough infection were set to be consistent with observations for the BNT162b2 booster clinical trial, after which waning antibody levels from Townsend et al [21] were paired with corresponding infection probabilities. Optimality of delayed boosting was then evaluated as the cumulative probability of infection spanning from the yearly optimal booster vaccination date following the infection through to the following yearly optimal booster vaccination date.

## RESULTS

**Figure 1.**
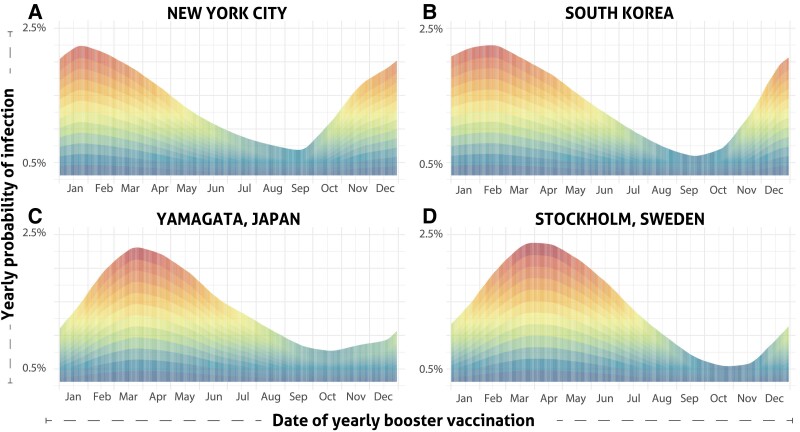
Yearly probability of infection (red [high] to blue [low]) based on yearly date of booster vaccination in New York (*A*), South Korea (*B*), Yamagata (*C*), and Stockholm (*D*).

Studies that have not incorporated the date of boosting into their analyses have found that annual boosters reduce the probability of infection by approximately 67% [19]. However, our analysis reveals that seasonality as well as the date of booster vaccination contribute substantially to the variance in efficacy (Figure 1). In general, each location exhibits an optimal date on which boosting offers a 3–4-fold increase in protection from infection over other times of the year, and a range of nearby dates that feature similar benefits. The timing of these periods varies substantially between locations (Figure 1; phenomenological equations, Supplementary Table 1). In New York City (Figure 1*A*), yearly booster vaccination on the 15th of September provides the lowest yearly probability of infection. This benefit diminishes with delay as the yearly probability of infection increases to a 3.6-fold higher booster vaccination administration on the least-effective date, 24th of January. Yearly booster vaccination dates later in the year than this least-effective date are increasingly beneficial up to the optimal date.

A pattern similar to that of New York is predicted for South Korea (Figure 1*B*; Supplementary Table 2), where the date with the lowest yearly probability of infection is on the 19th of September. Boosting in September in South Korea is predicted to confer a nearly 5-fold increase in protection relative to delaying boosting to January or February. For Yamagata, Japan (Figure 1*C*), the yearly booster vaccination date with the lowest probability of infection was slightly later, on the 18th of October. Yearly probabilities of infection in Stockholm, Sweden (Figure 1*D*), were similar across the year to those for Yamagata, supporting booster vaccination in October. In Stockholm, infection risks remain similar for yearly booster vaccination in November, then rapidly increase to a nearly 5-fold differential compared with a much less effective yearly booster vaccination administered in the spring.

**Figure 2.**
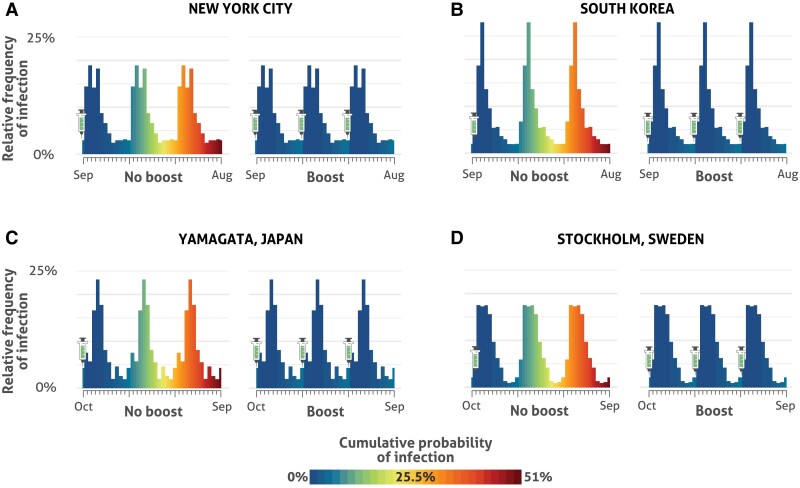
Cumulative probabilities of infection (lower to higher: blue/green/yellow/orange/red color gradient) in the year after booster vaccination occurring on the optimal date, in New York (*A*), South Korea (*B*), Yamagata (*C*), and Stockholm (*D*), when (left panels) booster vaccinated once, forgoing booster vaccination in the subsequent 2 years, and (right panels) booster vaccinated over a period of 3 years. The heights of the bars reflect projections of seasonal population-level incidence patterns under endemic conditions without incorporating a population-wide effect of booster vaccination—that is, assuming that low yearly global uptake [27] remains unchanged.

For other locations in the Northern Hemisphere, probabilities of infection throughout the year were often similar, reflecting a strong relationship that supports booster vaccine administration on a date that minimizes infection risk by preceding anticipated surges in SARS-CoV-2 incidence (Figure 2). On average across locales, the optimal yearly date of booster vaccine administration preceded the highest anticipated prevalence by 2.7 months (95% CI: 1.9–3.4 months) (Supplementary Figure 1). In areas for which seasonal infection trends were more muted, there was still benefit to optimizing booster vaccination timing, but the benefit was lesser (Supplementary Figure 2).

**Figure 3.**
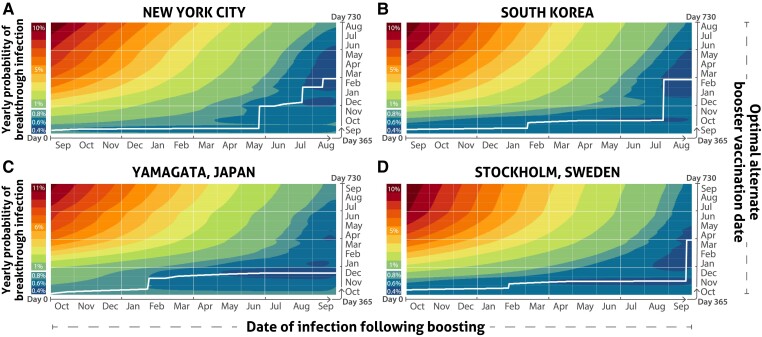
Probability of infection (lower to higher: blue/green/yellow/orange/red color gradient) upon delaying booster vaccination to each date of the year, in response to a breakthrough infection occurring in the interval between optimal booster vaccination dates in New York (*A*), South Korea (*B*), Yamagata (*C*), and Stockholm (*D*). Days 0 and 365 are analyzed as the estimated optimal yearly booster vaccination date. Infection will delay the optimal booster vaccination date (white line) beyond day 365 to an increasingly greater degree as the interval between the yearly optimal booster date (origin; see Figure 1) and the date of infection (*x* axis) increases.

The optimal timing of booster vaccination can be substantially altered by a breakthrough infection that occurs during the interval between yearly optimal booster vaccination dates (Figure 3; phenomenological equations, Supplementary Table 3). Indeed, in New York, a breakthrough infection occurring on any date between September through most of March has a practical equivalence of benefit to booster vaccination on the optimal yearly date of the 15th of September (Figure 3*A*; Supplementary Table 4). Delay of boosting begins to substantially accrue benefits after the middle of May (Figure 3*A*; Supplementary Table 4). For an individual infected in August—just before what would typically be the yearly optimal booster vaccination on the 15th of September—the next booster vaccination should be delayed and occur in late February, although similar benefits accrue to boosters obtained at any time between late January and June (Figure 3*A*; Supplementary Table 4).

Optimal booster timing was similarly delayed by breakthrough infection in other geographic locations (Figure 3*B*–3*D*; Supplementary Figure 2), and similarly varied depending on infection date (Supplementary Tables 5–13). Generally, individuals experiencing an unlikely, yet possible infection just subsequent to boosting on the optimal yearly booster vaccination date would benefit most by continuing to boost on the yearly optimal date. In contrast, those infected 6 months after the optimal yearly booster vaccination date will benefit most in terms of probability of infection over a yearlong span by delaying receipt of the next annual booster by several months. Importantly, for individuals who were infected just prior to the next optimal yearly booster vaccination date, the optimal delays of booster vaccination exceeded 9 months for nearly all locations.

## DISCUSSION

Here we have shown that the optimal timing of yearly booster vaccination in many locations within the temperate Northern Hemisphere is typically in the autumn to early winter, with moderate variance in timing across locations. In many cases, individuals who experience breakthrough infections can beneficially delay their upcoming booster. The amount of delay that provides the greatest benefit of booster vaccination is contingent on when the breakthrough infection occurs. Minimal delay in the next booster vaccination is warranted if an infection occurs shortly after a booster vaccination on the yearly optimal date. Most breakthrough infections are likely to occur long after booster vaccination [28], when substantial delay is most beneficial. Identification and communication of appropriate booster timing to public health authorities, vaccine providers, and recipients—along with guidance as to how to adapt that timing in response to infection—enables more effective booster vaccinations, timed to be cognizant of typical yearly incidence and individual infection history, and therefore more universally inclusive.

Our analysis is rigorously data-driven, but is based on a number of population- and location-specific datasets that enable us to quantify a broader generality. For instance, our yearly optimal booster vaccination dates are based on endemic seasonality of COVID-19 infection. However, infection trends for the near-term may not adhere to endemic seasonality and may be subject to greater variability in surge timing and intensity. Moreover, our analysis assumes that the typical endemic seasonality is applicable each year. Seasons of endemic respiratory viruses are known to vary in severity as well as to surge early or late in some years [29–31]. Indeed, the intervals between the optimal yearly booster vaccination dates and the peaks of monthly incidence were fairly consistent from location to location. This geographical consistency implies that a surge occurring earlier or later than usual at a location should be addressed by advancing or delaying the dates of booster administration in that location proportionately. Early and late surges are likely associated with factors such as temperature [32, 33], humidity [32], as well as environmentally driven shifts in human behavior [34]. Direct research on environmental correlates of surging infection for COVID-19 has been challenging due to pandemic dynamics of SARS-CoV-2, which have been strongly influenced by dramatic spatiotemporal shifts of immune status and of interventions [35, 36]. Therefore, accumulation of data regarding environmental correlates of endemic COVID-19 infection would empower the development of predictive seasonal incidence models that could, in turn, inform dynamic yearly recommendations regarding the timing of booster vaccination.

Our analyses are based on a typical healthy adult immune response to vaccination and infection and consequent waning of immunity. However, in modern human populations, immune status is highly heterogeneous [37, 38]. Factors such as immune-suppressive therapies, human immunodeficiency virus (HIV) infection, nutrition, pregnancy, as well as aging can all modulate the immune response. For individuals with compromised immune systems, it may be possible and beneficial to obtain additional booster vaccinations that can stimulate sufficient protection [20, 39, 40]. If restricted to a single booster vaccination per year, those with immune-compromising conditions would likely minimize their yearly probability of infection by timing their booster vaccination slightly later than the yearly optimal booster vaccination date determined for a typical healthy adult, ensuring that their lesser, shorter, peak protection spans the time at which peak seasonal infection occurs.

Booster vaccination has tremendous public health potential [20, 41]. However, uptake of COVID-19 booster vaccination by low proportions of residents has diminished its public health impact [27]. Our results show that appropriate timing of updated booster vaccination can provide a 3- to 5-fold improvement in the yearly protection provided. Perceptions of decreased susceptibility for infection, often stemming from known recent infections, are associated with decreased uptake of boosters [42]. For instance, healthcare workers who have been infected recently are unlikely to uptake, and can serve as unintentional drivers of vaccine hesitancy as they are viewed as models for public health responses by much of the general public [43]. Explicit guidance on how to adjust booster vaccination timing in response to infection—including in chronically exposed healthcare workers—acknowledges individual exposure history for a substantial portion of the population, and provides informed advice regarding their health.

Our analyses are based on Pfizer-BioNTech BNT1262b2 boosters under endemic conditions. However, changes in the efficacy between serially produced booster vaccines or between booster vaccines produced by different manufacturers may alter the relative level of protection provided at peak antibody level and during subsequent waning. Serial or alternate booster vaccines that provoke a lesser or greater antibody response will lead to yearly optimal booster dates and optimal delays following infection that are closer to or precede months in which incidence is high, respectively. Current booster vaccines with high levels of uptake, such as those manufactured by Moderna, induce antibody responses and protection from infection that is similar to booster vaccines from Pfizer-BioNTech [44].

The US CDC currently recommends that individuals who experience a breakthrough infection delay their booster uptake by up to 3 months [8]. This 3-month delay is appropriate for some midyear infections. However, this consistency is rather like a stopped clock that is correct at only certain times of the day: if a breakthrough infection occurred shortly after booster vaccination on the yearly optimal booster vaccination date, it is not optimal to delay the next booster at all beyond the next yearly optimal booster vaccination date. If a breakthrough infection occurred much later in the year following booster vaccination on the yearly optimal booster vaccination date—a more likely scenario—then it is optimal to delay the booster from the yearly optimal booster vaccination date to a much later date.

These results for COVID-19 provide the first continuous assessment of risk of infection with respect to annual booster vaccination for both people whose most recent exposure was a scheduled booster and for people whose most recent exposure was a breakthrough infection. For respiratory viruses with available vaccines, immunity wanes following vaccination, increasing the risk of breakthrough infection [45]. Robust analyses of optimal vaccine timing incorporating immuno-epidemiological inference [46] regarding numerous diseases including influenza and respiratory syncytial virus (RSV) are warranted, empowered by requisite collection of long-term longitudinal immunological cohort data from relevant individuals and long-term seasonal incidence data from diverse geographic locales. Together, immuno-epidemiological inference based on long-term infection data can play a substantial role in increasing the efficacy of booster vaccination, curtailing morbidity and mortality due to respiratory infectious disease.

## Supplementary Data

Supplementary materials are available at *Clinical Infectious Diseases* online. Consisting of data provided by the authors to benefit the reader, the posted materials are not copyedited and are the sole responsibility of the authors, so questions or comments should be addressed to the corresponding author.

## Supplementary Material

ciae559_Supplementary_Data

## Notes

***Author Contributions*.** J. P. T., H. B. H., and A. D. conceived the project and designed the study; H. B. H. performed formal analyses with guidance from J. P. T. and A. D.; J. P. T., H. B. H., and A. D. designed data visualizations; H. B. H. implemented data visualizations; A. D. and J. P. T. wrote the manuscript; and all authors reviewed the manuscript before submission. J. P. T. and A. D. were responsible for the decision to submit the manuscript. All authors had full access to all the data in the study and had final responsibility for the decision to submit for publication.

***Data availability.*** All data, Mathematica notebooks, imputed monthly proportions, and code underlying this study are publicly available on Zenodo: DOI: 10.5281/zenodo.11209811.

***Financial support*.** This work was supported by the National Science Foundation of the United States of America grants 2031204 (J. P. T. and A. D.) and 1918784 (J. P. T.), and with support from the University of North Carolina, Charlotte (to A. D.).
