# Supplementary material for: Optimal Annual COVID-19 Vaccine Boosting Dates Following Previous Booster Vaccination or Breakthrough Infection
Source: Clin Infect Dis. 2024 Nov 26;80(2):316–22. doi: 10.1093/cid/ciae559 (PMC11848277; doi:10.1093/cid/ciae559)
Supplement: ciae559_Supplementary_Data [file ciae559_supplementary_data.zip › TableS10_sweden.pdf]

Supplementary Table S10	Sweden
**Infection date**	**Following year optimal booster date**
14-Nov	14-Nov
15-Nov	14-Nov
16-Nov	14-Nov
17-Nov	14-Nov
18-Nov	14-Nov
19-Nov	15-Nov
20-Nov	15-Nov
21-Nov	15-Nov
22-Nov	15-Nov
23-Nov	15-Nov
24-Nov	15-Nov
25-Nov	15-Nov
26-Nov	15-Nov
27-Nov	15-Nov
28-Nov	16-Nov
29-Nov	16-Nov
30-Nov	16-Nov
1-Dec	16-Nov
2-Dec	16-Nov
3-Dec	16-Nov
4-Dec	16-Nov
5-Dec	16-Nov
6-Dec	16-Nov
7-Dec	16-Nov
8-Dec	16-Nov
9-Dec	16-Nov
10-Dec	16-Nov
11-Dec	16-Nov
12-Dec	16-Nov
13-Dec	16-Nov
14-Dec	16-Nov
15-Dec	16-Nov
16-Dec	16-Nov
17-Dec	16-Nov
18-Dec	16-Nov
19-Dec	16-Nov
20-Dec	16-Nov
21-Dec	16-Nov
22-Dec	16-Nov
23-Dec	16-Nov
24-Dec	16-Nov

25-Dec	16-Nov
26-Dec	16-Nov
27-Dec	16-Nov
28-Dec	16-Nov
29-Dec	16-Nov
30-Dec	16-Nov
31-Dec	16-Nov
1-Jan	16-Nov
2-Jan	16-Nov
3-Jan	16-Nov
4-Jan	16-Nov
5-Jan	16-Nov
6-Jan	16-Nov
7-Jan	16-Nov
8-Jan	17-Nov
9-Jan	17-Nov
10-Jan	17-Nov
11-Jan	17-Nov
12-Jan	17-Nov
13-Jan	17-Nov
14-Jan	17-Nov
15-Jan	17-Nov
16-Jan	17-Nov
17-Jan	17-Nov
18-Jan	17-Nov
19-Jan	17-Nov
20-Jan	17-Nov
21-Jan	17-Nov
22-Jan	17-Nov
23-Jan	17-Nov
24-Jan	17-Nov
25-Jan	17-Nov
26-Jan	17-Nov
27-Jan	17-Nov
28-Jan	17-Nov
29-Jan	17-Nov
30-Jan	17-Nov
31-Jan	17-Nov
1-Feb	17-Nov
2-Feb	17-Nov
3-Feb	17-Nov
4-Feb	17-Nov
5-Feb	17-Nov

6-Feb	17-Nov
7-Feb	17-Nov
8-Feb	17-Nov
9-Feb	17-Nov
10-Feb	17-Nov
11-Feb	17-Nov
12-Feb	17-Nov
13-Feb	17-Nov
14-Feb	17-Nov
15-Feb	17-Nov
16-Feb	17-Nov
17-Feb	17-Nov
18-Feb	17-Nov
19-Feb	17-Nov
20-Feb	17-Nov
21-Feb	17-Nov
22-Feb	17-Nov
23-Feb	17-Nov
24-Feb	17-Nov
25-Feb	17-Nov
26-Feb	17-Nov
27-Feb	17-Nov
28-Feb	17-Nov
1-Mar	18-Nov
2-Mar	18-Nov
3-Mar	18-Nov
4-Mar	18-Nov
5-Mar	18-Nov
6-Mar	18-Nov
7-Mar	18-Nov
8-Mar	18-Nov
9-Mar	18-Nov
10-Mar	18-Nov
11-Mar	18-Nov
12-Mar	18-Nov
13-Mar	18-Nov
14-Mar	18-Nov
15-Mar	18-Nov
16-Mar	18-Nov
17-Mar	18-Nov
18-Mar	18-Nov
19-Mar	18-Nov
20-Mar	18-Nov

21-Mar	18-Nov
22-Mar	18-Nov
23-Mar	18-Nov
24-Mar	18-Nov
25-Mar	18-Nov
26-Mar	18-Nov
27-Mar	18-Nov
28-Mar	18-Nov
29-Mar	18-Nov
30-Mar	18-Nov
31-Mar	18-Nov
1-Apr	18-Nov
2-Apr	18-Nov
3-Apr	18-Nov
4-Apr	18-Nov
5-Apr	18-Nov
6-Apr	18-Nov
7-Apr	18-Nov
8-Apr	18-Nov
9-Apr	18-Nov
10-Apr	18-Nov
11-Apr	18-Nov
12-Apr	18-Nov
13-Apr	18-Nov
14-Apr	18-Nov
15-Apr	18-Nov
16-Apr	18-Nov
17-Apr	18-Nov
18-Apr	18-Nov
19-Apr	18-Nov
20-Apr	18-Nov
21-Apr	18-Nov
22-Apr	18-Nov
23-Apr	18-Nov
24-Apr	18-Nov
25-Apr	18-Nov
26-Apr	18-Nov
27-Apr	18-Nov
28-Apr	18-Nov
29-Apr	18-Nov
30-Apr	18-Nov
1-May	18-Nov
2-May	18-Nov

3-May	18-Nov
4-May	18-Nov
5-May	18-Nov
6-May	18-Nov
7-May	18-Nov
8-May	18-Nov
9-May	18-Nov
10-May	18-Nov
11-May	18-Nov
12-May	18-Nov
13-May	18-Nov
14-May	18-Nov
15-May	18-Nov
16-May	18-Nov
17-May	18-Nov
18-May	18-Nov
19-May	18-Nov
20-May	18-Nov
21-May	18-Nov
22-May	19-Nov
23-May	19-Nov
24-May	19-Nov
25-May	19-Nov
26-May	19-Nov
27-May	19-Nov
28-May	19-Nov
29-May	19-Nov
30-May	19-Nov
31-May	19-Nov
1-Jun	19-Nov
2-Jun	19-Nov
3-Jun	19-Nov
4-Jun	19-Nov
5-Jun	19-Nov
6-Jun	19-Nov
7-Jun	19-Nov
8-Jun	19-Nov
9-Jun	19-Nov
10-Jun	19-Nov
11-Jun	19-Nov
12-Jun	19-Nov
13-Jun	19-Nov
14-Jun	19-Nov

15-Jun	19-Nov
16-Jun	19-Nov
17-Jun	19-Nov
18-Jun	19-Nov
19-Jun	19-Nov
20-Jun	19-Nov
21-Jun	19-Nov
22-Jun	19-Nov
23-Jun	19-Nov
24-Jun	19-Nov
25-Jun	19-Nov
26-Jun	19-Nov
27-Jun	19-Nov
28-Jun	19-Nov
29-Jun	19-Nov
30-Jun	19-Nov
1-Jul	19-Nov
2-Jul	19-Nov
3-Jul	19-Nov
4-Jul	19-Nov
5-Jul	19-Nov
6-Jul	19-Nov
7-Jul	19-Nov
8-Jul	19-Nov
9-Jul	19-Nov
10-Jul	19-Nov
11-Jul	19-Nov
12-Jul	22-Dec
13-Jul	22-Dec
14-Jul	22-Dec
15-Jul	23-Dec
16-Jul	23-Dec
17-Jul	14-Jan
18-Jul	15-Jan
19-Jul	15-Jan
20-Jul	28-Feb
21-Jul	28-Feb
22-Jul	28-Feb
23-Jul	28-Feb
24-Jul	28-Feb
25-Jul	28-Feb
26-Jul	28-Feb
27-Jul	28-Feb

28-Jul	31-Mar
29-Jul	31-Mar
30-Jul	31-Mar
31-Jul	31-Mar
1-Aug	31-Mar
2-Aug	31-Mar
3-Aug	31-Mar
4-Aug	31-Mar
5-Aug	31-Mar
6-Aug	31-Mar
7-Aug	31-Mar
8-Aug	31-Mar
9-Aug	31-Mar
10-Aug	31-Mar
11-Aug	31-Mar
12-Aug	31-Mar
13-Aug	31-Mar
14-Aug	31-Mar
15-Aug	31-Mar
16-Aug	31-Mar
17-Aug	31-Mar
18-Aug	31-Mar
19-Aug	31-Mar
20-Aug	31-Mar
21-Aug	31-Mar
22-Aug	31-Mar
23-Aug	31-Mar
24-Aug	31-Mar
25-Aug	31-Mar
26-Aug	31-Mar
27-Aug	31-Mar
28-Aug	31-Mar
29-Aug	31-Mar
30-Aug	31-Mar
31-Aug	31-Mar
1-Sep	31-Mar
2-Sep	31-Mar
3-Sep	31-Mar
4-Sep	31-Mar
5-Sep	31-Mar
6-Sep	31-Mar
7-Sep	31-Mar
8-Sep	31-Mar

9-Sep	1-Apr
10-Sep	1-Apr
11-Sep	2-Apr
12-Sep	2-Apr
13-Sep	3-Apr
14-Sep	3-Apr
15-Sep	4-Apr
16-Sep	4-Apr
17-Sep	5-Apr
18-Sep	5-Apr
19-Sep	6-Apr
20-Sep	6-Apr
21-Sep	7-Apr
22-Sep	7-Apr
23-Sep	7-Apr
24-Sep	8-Apr
25-Sep	8-Apr
26-Sep	9-Apr
27-Sep	9-Apr
28-Sep	9-Apr
29-Sep	10-Apr
30-Sep	10-Apr
1-Oct	11-Apr
2-Oct	11-Apr
3-Oct	11-Apr
4-Oct	12-Apr
5-Oct	12-Apr
6-Oct	12-Apr
7-Oct	13-Apr
8-Oct	13-Apr
9-Oct	14-Apr
10-Oct	14-Apr
11-Oct	14-Apr
12-Oct	15-Apr
13-Oct	15-Apr
14-Oct	16-Apr
15-Oct	16-Apr
16-Oct	16-Apr
17-Oct	16-Apr
18-Oct	16-Apr
19-Oct	16-Apr
20-Oct	17-Apr
21-Oct	17-Apr

22-Oct	17-Apr
23-Oct	17-Apr
24-Oct	17-Apr
25-Oct	17-Apr
26-Oct	18-Apr
27-Oct	18-Apr
28-Oct	18-Apr
29-Oct	18-Apr
30-Oct	18-Apr
31-Oct	18-Apr
1-Nov	18-Apr
2-Nov	19-Apr
3-Nov	19-Apr
4-Nov	19-Apr
5-Nov	19-Apr
6-Nov	19-Apr
7-Nov	19-Apr
8-Nov	20-Apr
9-Nov	20-Apr
10-Nov	20-Apr
11-Nov	20-Apr
12-Nov	20-Apr
13-Nov	20-Apr
