# Supplementary material for: Optimal Annual COVID-19 Vaccine Boosting Dates Following Previous Booster Vaccination or Breakthrough Infection
Source: Clin Infect Dis. 2024 Nov 26;80(2):316–22. doi: 10.1093/cid/ciae559 (PMC11848277; doi:10.1093/cid/ciae559)
Supplement: ciae559_Supplementary_Data [file ciae559_supplementary_data.zip › TableS11_israel.pdf]

Supplementary Table S11	Israel
**Infection date**	**Following year optimal booster date**
21-Feb	21-Feb
22-Feb	21-Feb
23-Feb	21-Feb
24-Feb	21-Feb
25-Feb	21-Feb
26-Feb	21-Feb
27-Feb	21-Feb
28-Feb	21-Feb
1-Mar	21-Feb
2-Mar	21-Feb
3-Mar	21-Feb
4-Mar	21-Feb
5-Mar	21-Feb
6-Mar	21-Feb
7-Mar	21-Feb
8-Mar	21-Feb
9-Mar	21-Feb
10-Mar	21-Feb
11-Mar	21-Feb
12-Mar	21-Feb
13-Mar	21-Feb
14-Mar	21-Feb
15-Mar	21-Feb
16-Mar	21-Feb
17-Mar	21-Feb
18-Mar	21-Feb
19-Mar	21-Feb
20-Mar	21-Feb
21-Mar	21-Feb
22-Mar	21-Feb
23-Mar	21-Feb
24-Mar	21-Feb
25-Mar	21-Feb
26-Mar	21-Feb
27-Mar	21-Feb
28-Mar	21-Feb
29-Mar	21-Feb
30-Mar	21-Feb
31-Mar	21-Feb
1-Apr	21-Feb
2-Apr	21-Feb

3-Apr	21-Feb
4-Apr	21-Feb
5-Apr	21-Feb
6-Apr	21-Feb
7-Apr	21-Feb
8-Apr	21-Feb
9-Apr	21-Feb
10-Apr	21-Feb
11-Apr	21-Feb
12-Apr	21-Feb
13-Apr	21-Feb
14-Apr	21-Feb
15-Apr	21-Feb
16-Apr	21-Feb
17-Apr	21-Feb
18-Apr	21-Feb
19-Apr	21-Feb
20-Apr	21-Feb
21-Apr	21-Feb
22-Apr	21-Feb
23-Apr	21-Feb
24-Apr	21-Feb
25-Apr	21-Feb
26-Apr	21-Feb
27-Apr	21-Feb
28-Apr	21-Feb
29-Apr	21-Feb
30-Apr	21-Feb
1-May	21-Feb
2-May	21-Feb
3-May	21-Feb
4-May	21-Feb
5-May	21-Feb
6-May	21-Feb
7-May	21-Feb
8-May	21-Feb
9-May	21-Feb
10-May	21-Feb
11-May	21-Feb
12-May	21-Feb
13-May	21-Feb
14-May	21-Feb
15-May	21-Feb

16-May	21-Feb
17-May	21-Feb
18-May	21-Feb
19-May	21-Feb
20-May	21-Feb
21-May	21-Feb
22-May	21-Feb
23-May	21-Feb
24-May	21-Feb
25-May	21-Feb
26-May	21-Mar
27-May	23-Mar
28-May	24-Mar
29-May	25-Mar
30-May	26-Mar
31-May	27-Mar
1-Jun	28-Mar
2-Jun	29-Mar
3-Jun	30-Mar
4-Jun	31-Mar
5-Jun	31-Mar
6-Jun	31-Mar
7-Jun	31-Mar
8-Jun	31-Mar
9-Jun	31-Mar
10-Jun	31-Mar
11-Jun	31-Mar
12-Jun	31-Mar
13-Jun	31-Mar
14-Jun	1-Apr
15-Jun	1-Apr
16-Jun	2-Apr
17-Jun	2-Apr
18-Jun	3-Apr
19-Jun	3-Apr
20-Jun	4-Apr
21-Jun	4-Apr
22-Jun	5-Apr
23-Jun	5-Apr
24-Jun	6-Apr
25-Jun	6-Apr
26-Jun	6-Apr
27-Jun	6-Apr

28-Jun	7-Apr
29-Jun	7-Apr
30-Jun	7-Apr
1-Jul	7-Apr
2-Jul	8-Apr
3-Jul	8-Apr
4-Jul	8-Apr
5-Jul	8-Apr
6-Jul	9-Apr
7-Jul	9-Apr
8-Jul	9-Apr
9-Jul	9-Apr
10-Jul	10-Apr
11-Jul	10-Apr
12-Jul	10-Apr
13-Jul	10-Apr
14-Jul	11-Apr
15-Jul	11-Apr
16-Jul	11-Apr
17-Jul	11-Apr
18-Jul	12-Apr
19-Jul	12-Apr
20-Jul	12-Apr
21-Jul	12-Apr
22-Jul	13-Apr
23-Jul	13-Apr
24-Jul	13-Apr
25-Jul	13-Apr
26-Jul	14-Apr
27-Jul	14-Apr
28-Jul	14-Apr
29-Jul	14-Apr
30-Jul	14-Apr
31-Jul	15-Apr
1-Aug	15-Apr
2-Aug	15-Apr
3-Aug	15-Apr
4-Aug	16-Apr
5-Aug	16-Apr
6-Aug	16-Apr
7-Aug	16-Apr
8-Aug	16-Apr
9-Aug	16-Apr

10-Aug	16-Apr
11-Aug	16-Apr
12-Aug	16-Apr
13-Aug	16-Apr
14-Aug	16-Apr
15-Aug	16-Apr
16-Aug	16-Apr
17-Aug	16-Apr
18-Aug	16-Apr
19-Aug	16-Apr
20-Aug	17-Apr
21-Aug	17-Apr
22-Aug	17-Apr
23-Aug	17-Apr
24-Aug	17-Apr
25-Aug	17-Apr
26-Aug	17-Apr
27-Aug	10-Jul
28-Aug	10-Jul
29-Aug	10-Jul
30-Aug	10-Jul
31-Aug	10-Jul
1-Sep	10-Jul
2-Sep	10-Jul
3-Sep	10-Jul
4-Sep	10-Jul
5-Sep	10-Jul
6-Sep	10-Jul
7-Sep	10-Jul
8-Sep	10-Jul
9-Sep	10-Jul
10-Sep	10-Jul
11-Sep	10-Jul
12-Sep	10-Jul
13-Sep	10-Jul
14-Sep	11-Jul
15-Sep	11-Jul
16-Sep	11-Jul
17-Sep	11-Jul
18-Sep	11-Jul
19-Sep	11-Jul
20-Sep	11-Jul
21-Sep	11-Jul

22-Sep	11-Jul
23-Sep	11-Jul
24-Sep	11-Jul
25-Sep	11-Jul
26-Sep	11-Jul
27-Sep	11-Jul
28-Sep	11-Jul
29-Sep	11-Jul
30-Sep	11-Jul
1-Oct	11-Jul
2-Oct	11-Jul
3-Oct	11-Jul
4-Oct	11-Jul
5-Oct	11-Jul
6-Oct	11-Jul
7-Oct	11-Jul
8-Oct	11-Jul
9-Oct	11-Jul
10-Oct	11-Jul
11-Oct	11-Jul
12-Oct	11-Jul
13-Oct	11-Jul
14-Oct	12-Jul
15-Oct	12-Jul
16-Oct	12-Jul
17-Oct	12-Jul
18-Oct	12-Jul
19-Oct	12-Jul
20-Oct	12-Jul
21-Oct	12-Jul
22-Oct	12-Jul
23-Oct	12-Jul
24-Oct	12-Jul
25-Oct	12-Jul
26-Oct	12-Jul
27-Oct	12-Jul
28-Oct	12-Jul
29-Oct	12-Jul
30-Oct	12-Jul
31-Oct	12-Jul
1-Nov	12-Jul
2-Nov	12-Jul
3-Nov	12-Jul

4-Nov	12-Jul
5-Nov	12-Jul
6-Nov	12-Jul
7-Nov	12-Jul
8-Nov	12-Jul
9-Nov	12-Jul
10-Nov	12-Jul
11-Nov	12-Jul
12-Nov	12-Jul
13-Nov	12-Jul
14-Nov	12-Jul
15-Nov	12-Jul
16-Nov	12-Jul
17-Nov	12-Jul
18-Nov	12-Jul
19-Nov	12-Jul
20-Nov	12-Jul
21-Nov	12-Jul
22-Nov	13-Jul
23-Nov	13-Jul
24-Nov	13-Jul
25-Nov	13-Jul
26-Nov	13-Jul
27-Nov	13-Jul
28-Nov	13-Jul
29-Nov	13-Jul
30-Nov	13-Jul
1-Dec	13-Jul
2-Dec	13-Jul
3-Dec	13-Jul
4-Dec	13-Jul
5-Dec	13-Jul
6-Dec	13-Jul
7-Dec	13-Jul
8-Dec	13-Jul
9-Dec	13-Jul
10-Dec	13-Jul
11-Dec	13-Jul
12-Dec	13-Jul
13-Dec	13-Jul
14-Dec	13-Jul
15-Dec	13-Jul
16-Dec	13-Jul

17-Dec	13-Jul
18-Dec	13-Jul
19-Dec	13-Jul
20-Dec	13-Jul
21-Dec	13-Jul
22-Dec	13-Jul
23-Dec	13-Jul
24-Dec	13-Jul
25-Dec	13-Jul
26-Dec	13-Jul
27-Dec	13-Jul
28-Dec	13-Jul
29-Dec	13-Jul
30-Dec	13-Jul
31-Dec	13-Jul
1-Jan	13-Jul
2-Jan	13-Jul
3-Jan	13-Jul
4-Jan	13-Jul
5-Jan	13-Jul
6-Jan	13-Jul
7-Jan	13-Jul
8-Jan	13-Jul
9-Jan	13-Jul
10-Jan	13-Jul
11-Jan	13-Jul
12-Jan	13-Jul
13-Jan	13-Jul
14-Jan	13-Jul
15-Jan	13-Jul
16-Jan	13-Jul
17-Jan	14-Jul
18-Jan	14-Jul
19-Jan	14-Jul
20-Jan	14-Jul
21-Jan	14-Jul
22-Jan	14-Jul
23-Jan	14-Jul
24-Jan	14-Jul
25-Jan	14-Jul
26-Jan	14-Jul
27-Jan	14-Jul
28-Jan	14-Jul

29-Jan	14-Jul
30-Jan	14-Jul
31-Jan	14-Jul
1-Feb	14-Jul
2-Feb	14-Jul
3-Feb	14-Jul
4-Feb	14-Jul
5-Feb	14-Jul
6-Feb	14-Jul
7-Feb	14-Jul
8-Feb	14-Jul
9-Feb	14-Jul
10-Feb	14-Jul
11-Feb	14-Jul
12-Feb	14-Jul
13-Feb	14-Jul
14-Feb	14-Jul
15-Feb	14-Jul
16-Feb	14-Jul
17-Feb	14-Jul
18-Feb	14-Jul
19-Feb	14-Jul
20-Feb	14-Jul
