# Supplementary material for: Optimal Annual COVID-19 Vaccine Boosting Dates Following Previous Booster Vaccination or Breakthrough Infection
Source: Clin Infect Dis. 2024 Nov 26;80(2):316–22. doi: 10.1093/cid/ciae559 (PMC11848277; doi:10.1093/cid/ciae559)
Supplement: ciae559_Supplementary_Data [file ciae559_supplementary_data.zip › TableS13_stockholm.pdf]

Supplementary Table S13 Stockholm

Infection date	Following year optimal booster date
21-Oct	21-Oct
22-Oct	21-Oct
23-Oct	21-Oct
24-Oct	21-Oct
25-Oct	21-Oct
26-Oct	21-Oct
27-Oct	21-Oct
28-Oct	21-Oct
29-Oct	21-Oct
30-Oct	22-Oct
31-Oct	22-Oct
1-Nov	22-Oct
2-Nov	22-Oct
3-Nov	22-Oct
4-Nov	22-Oct
5-Nov	22-Oct
6-Nov	22-Oct
7-Nov	22-Oct
8-Nov	22-Oct
9-Nov	22-Oct
10-Nov	22-Oct
11-Nov	22-Oct
12-Nov	22-Oct
13-Nov	22-Oct
14-Nov	22-Oct
15-Nov	22-Oct
16-Nov	22-Oct
17-Nov	22-Oct
18-Nov	22-Oct
19-Nov	22-Oct
20-Nov	22-Oct
21-Nov	23-Oct
22-Nov	23-Oct
23-Nov	23-Oct
24-Nov	23-Oct
25-Nov	23-Oct
26-Nov	23-Oct
27-Nov	23-Oct
28-Nov	23-Oct
29-Nov	23-Oct
30-Nov	23-Oct

1-Dec	23-Oct
2-Dec	23-Oct
3-Dec	23-Oct
4-Dec	23-Oct
5-Dec	23-Oct
6-Dec	23-Oct
7-Dec	23-Oct
8-Dec	23-Oct
9-Dec	23-Oct
10-Dec	23-Oct
11-Dec	23-Oct
12-Dec	23-Oct
13-Dec	23-Oct
14-Dec	23-Oct
15-Dec	24-Oct
16-Dec	24-Oct
17-Dec	24-Oct
18-Dec	24-Oct
19-Dec	24-Oct
20-Dec	24-Oct
21-Dec	24-Oct
22-Dec	24-Oct
23-Dec	24-Oct
24-Dec	24-Oct
25-Dec	24-Oct
26-Dec	24-Oct
27-Dec	24-Oct
28-Dec	24-Oct
29-Dec	24-Oct
30-Dec	24-Oct
31-Dec	24-Oct
1-Jan	24-Oct
2-Jan	24-Oct
3-Jan	24-Oct
4-Jan	25-Oct
5-Jan	25-Oct
6-Jan	25-Oct
7-Jan	25-Oct
8-Jan	25-Oct
9-Jan	25-Oct
10-Jan	25-Oct
11-Jan	25-Oct
12-Jan	25-Oct

13-Jan	25-Oct
14-Jan	25-Oct
15-Jan	25-Oct
16-Jan	25-Oct
17-Jan	25-Oct
18-Jan	25-Oct
19-Jan	25-Oct
20-Jan	25-Oct
21-Jan	25-Oct
22-Jan	25-Oct
23-Jan	25-Oct
24-Jan	25-Oct
25-Jan	7-Nov
26-Jan	7-Nov
27-Jan	7-Nov
28-Jan	8-Nov
29-Jan	8-Nov
30-Jan	8-Nov
31-Jan	8-Nov
1-Feb	8-Nov
2-Feb	8-Nov
3-Feb	8-Nov
4-Feb	9-Nov
5-Feb	9-Nov
6-Feb	9-Nov
7-Feb	9-Nov
8-Feb	9-Nov
9-Feb	9-Nov
10-Feb	9-Nov
11-Feb	9-Nov
12-Feb	10-Nov
13-Feb	10-Nov
14-Feb	10-Nov
15-Feb	10-Nov
16-Feb	10-Nov
17-Feb	10-Nov
18-Feb	10-Nov
19-Feb	10-Nov
20-Feb	11-Nov
21-Feb	11-Nov
22-Feb	11-Nov
23-Feb	11-Nov
24-Feb	11-Nov

25-Feb	11-Nov
26-Feb	11-Nov
27-Feb	11-Nov
28-Feb	12-Nov
1-Mar	12-Nov
2-Mar	12-Nov
3-Mar	12-Nov
4-Mar	12-Nov
5-Mar	12-Nov
6-Mar	12-Nov
7-Mar	12-Nov
8-Mar	12-Nov
9-Mar	13-Nov
10-Mar	13-Nov
11-Mar	13-Nov
12-Mar	13-Nov
13-Mar	13-Nov
14-Mar	13-Nov
15-Mar	13-Nov
16-Mar	13-Nov
17-Mar	13-Nov
18-Mar	14-Nov
19-Mar	14-Nov
20-Mar	14-Nov
21-Mar	14-Nov
22-Mar	14-Nov
23-Mar	14-Nov
24-Mar	14-Nov
25-Mar	14-Nov
26-Mar	14-Nov
27-Mar	14-Nov
28-Mar	15-Nov
29-Mar	15-Nov
30-Mar	15-Nov
31-Mar	15-Nov
1-Apr	15-Nov
2-Apr	15-Nov
3-Apr	15-Nov
4-Apr	15-Nov
5-Apr	15-Nov
6-Apr	15-Nov
7-Apr	16-Nov
8-Apr	16-Nov

9-Apr	16-Nov
10-Apr	16-Nov
11-Apr	16-Nov
12-Apr	16-Nov
13-Apr	16-Nov
14-Apr	16-Nov
15-Apr	16-Nov
16-Apr	16-Nov
17-Apr	16-Nov
18-Apr	16-Nov
19-Apr	16-Nov
20-Apr	16-Nov
21-Apr	16-Nov
22-Apr	16-Nov
23-Apr	16-Nov
24-Apr	16-Nov
25-Apr	16-Nov
26-Apr	16-Nov
27-Apr	16-Nov
28-Apr	16-Nov
29-Apr	16-Nov
30-Apr	16-Nov
1-May	16-Nov
2-May	16-Nov
3-May	16-Nov
4-May	16-Nov
5-May	16-Nov
6-May	16-Nov
7-May	16-Nov
8-May	16-Nov
9-May	16-Nov
10-May	16-Nov
11-May	16-Nov
12-May	16-Nov
13-May	16-Nov
14-May	16-Nov
15-May	16-Nov
16-May	16-Nov
17-May	16-Nov
18-May	16-Nov
19-May	16-Nov
20-May	16-Nov
21-May	16-Nov

22-May	16-Nov
23-May	16-Nov
24-May	16-Nov
25-May	16-Nov
26-May	16-Nov
27-May	16-Nov
28-May	16-Nov
29-May	16-Nov
30-May	16-Nov
31-May	16-Nov
1-Jun	16-Nov
2-Jun	16-Nov
3-Jun	16-Nov
4-Jun	16-Nov
5-Jun	16-Nov
6-Jun	16-Nov
7-Jun	16-Nov
8-Jun	16-Nov
9-Jun	16-Nov
10-Jun	16-Nov
11-Jun	16-Nov
12-Jun	17-Nov
13-Jun	17-Nov
14-Jun	17-Nov
15-Jun	17-Nov
16-Jun	17-Nov
17-Jun	17-Nov
18-Jun	17-Nov
19-Jun	17-Nov
20-Jun	17-Nov
21-Jun	17-Nov
22-Jun	17-Nov
23-Jun	17-Nov
24-Jun	17-Nov
25-Jun	17-Nov
26-Jun	17-Nov
27-Jun	17-Nov
28-Jun	17-Nov
29-Jun	17-Nov
30-Jun	17-Nov
1-Jul	17-Nov
2-Jul	17-Nov
3-Jul	17-Nov

4-Jul	17-Nov
5-Jul	17-Nov
6-Jul	17-Nov
7-Jul	17-Nov
8-Jul	17-Nov
9-Jul	17-Nov
10-Jul	17-Nov
11-Jul	17-Nov
12-Jul	17-Nov
13-Jul	17-Nov
14-Jul	17-Nov
15-Jul	17-Nov
16-Jul	17-Nov
17-Jul	17-Nov
18-Jul	17-Nov
19-Jul	17-Nov
20-Jul	17-Nov
21-Jul	17-Nov
22-Jul	17-Nov
23-Jul	17-Nov
24-Jul	17-Nov
25-Jul	17-Nov
26-Jul	17-Nov
27-Jul	17-Nov
28-Jul	17-Nov
29-Jul	17-Nov
30-Jul	17-Nov
31-Jul	17-Nov
1-Aug	17-Nov
2-Aug	17-Nov
3-Aug	17-Nov
4-Aug	17-Nov
5-Aug	17-Nov
6-Aug	17-Nov
7-Aug	17-Nov
8-Aug	17-Nov
9-Aug	17-Nov
10-Aug	17-Nov
11-Aug	17-Nov
12-Aug	17-Nov
13-Aug	17-Nov
14-Aug	17-Nov
15-Aug	17-Nov

16-Aug	17-Nov
17-Aug	17-Nov
18-Aug	17-Nov
19-Aug	17-Nov
20-Aug	17-Nov
21-Aug	17-Nov
22-Aug	17-Nov
23-Aug	17-Nov
24-Aug	17-Nov
25-Aug	17-Nov
26-Aug	17-Nov
27-Aug	17-Nov
28-Aug	17-Nov
29-Aug	17-Nov
30-Aug	17-Nov
31-Aug	17-Nov
1-Sep	17-Nov
2-Sep	17-Nov
3-Sep	17-Nov
4-Sep	17-Nov
5-Sep	17-Nov
6-Sep	17-Nov
7-Sep	17-Nov
8-Sep	21-Jan
9-Sep	22-Jan
10-Sep	22-Jan
11-Sep	23-Jan
12-Sep	23-Jan
13-Sep	23-Jan
14-Sep	24-Jan
15-Sep	24-Jan
16-Sep	31-Mar
17-Sep	31-Mar
18-Sep	31-Mar
19-Sep	31-Mar
20-Sep	31-Mar
21-Sep	31-Mar
22-Sep	31-Mar
23-Sep	31-Mar
24-Sep	31-Mar
25-Sep	31-Mar
26-Sep	31-Mar
27-Sep	31-Mar

28-Sep	31-Mar
29-Sep	31-Mar
30-Sep	31-Mar
1-Oct	31-Mar
2-Oct	31-Mar
3-Oct	31-Mar
4-Oct	31-Mar
5-Oct	31-Mar
6-Oct	31-Mar
7-Oct	31-Mar
8-Oct	31-Mar
9-Oct	31-Mar
10-Oct	31-Mar
11-Oct	31-Mar
12-Oct	31-Mar
13-Oct	31-Mar
14-Oct	31-Mar
15-Oct	30-Apr
16-Oct	30-Apr
17-Oct	30-Apr
18-Oct	30-Apr
19-Oct	30-Apr
20-Oct	30-Apr
