# Supplementary material for: Optimal Annual COVID-19 Vaccine Boosting Dates Following Previous Booster Vaccination or Breakthrough Infection
Source: Clin Infect Dis. 2024 Nov 26;80(2):316–22. doi: 10.1093/cid/ciae559 (PMC11848277; doi:10.1093/cid/ciae559)
Supplement: ciae559_Supplementary_Data [file ciae559_supplementary_data.zip › TableS1_equations1.pdf]

Table S1. Phenomenological equations for Figure 1

Location	Equation for yearly probability of infection based on yearly date of booster vaccination
New York	$0.02 + 1.77 \times 10^{-4}x - 4.04 \times 10^{-7}x^2 - 4.31 \times 10^{-9}x^3 + 2.90 \times 10^{-11}x^4 - 3.98 \times 10^{-14}x^5$
Stockholm	$0.01 + 2.21 \times 10^{-6}x - 1.44 \times 10^{-6}x^2 + 2.52 \times 10^{-10}x^3 + 7.77 \times 10^{-12}x^4 - 5.97 \times 10^{-15}x^5$
South Korea	$0.02 - 2.86 \times 10^{-6}x - 5.75 \times 10^{-7}x^2 + 6.73 \times 10^{-10}x^3 + 1.81 \times 10^{-12}x^4 + 2.26 \times 10^{-15}x^5$
Edinburgh	$0.01 + 2.20 \times 10^{-4}x - 7.55 \times 10^{-7}x^2 - 8.44 \times 10^{-9}x^3 + 4.30 \times 10^{-11}x^4 - 5.10 \times 10^{-14}x^5$
Yamagata	$0.01 + 3.53 \times 10^{-4}x - 2.66 \times 10^{-6}x^2 + 3.82 \times 10^{-9}x^3 + 9.83 \times 10^{-12}x^4 - 2.07 \times 10^{-14}x^5$
Nepal	$0.01 + 2.21 \times 10^{-4}x - 2.20 \times 10^{-6}x^2 + 6.58 \times 10^{-9}x^3 - 5.05 \times 10^{-12}x^4 - 2.74 \times 10^{-15}x^5$
Guangzhou	$0.02 + 1.63 \times 10^{-4}x - 1.41 \times 10^{-6}x^2 + 1.33 \times 10^{-9}x^3 + 6.53 \times 10^{-12}x^4 - 7.75 \times 10^{-15}x^5$
Netherlands	$0.01 + 2.09 \times 10^{-5}x + 8.16 \times 10^{-7}x^2 - 8.85 \times 10^{-9}x^3 + 2.71 \times 10^{-11}x^4 - 2.58 \times 10^{-14}x^5$
Gothenburg	$0.01 + 3.15 \times 10^{-4}x - 3.91 \times 10^{-6}x^2 + 1.90 \times 10^{-8}x^3 - 4.68 \times 10^{-11}x^4 + 4.81 \times 10^{-14}x^5$
Norway	$0.02 + 1.63 \times 10^{-4}x - 1.41 \times 10^{-6}x^2 + 1.33 \times 10^{-9}x^3 + 6.53 \times 10^{-12}x^4 - 7.75 \times 10^{-15}x^5$
Israel	$0.02 + 1.24 \times 10^{-4}x - 4.28 \times 10^{-5}x^2 + 4.32 \times 10^{-6}x^3 - 2.33 \times 10^{-7}x^4 + 7.36 \times 10^{-9}x^5 - 1.48 \times 10^{-10}x^6 + 2.00 \times 10^{-12}x^7 - 1.90 \times 10^{-14}x^8 + 1.30 \times 10^{-16}x^9 - 6.41 \times 10^{-19}x^{10} + 2.31 \times 10^{-21}x^{11} - 5.99 \times 10^{-24}x^{12} + 1.09 \times 10^{-26}x^{13} - 1.32 \times 10^{-29}x^{14} + 9.55 \times 10^{-33}x^{15} - 3.13 \times 10^{-36}x^{16}$
