# Supplementary material for: Optimal Annual COVID-19 Vaccine Boosting Dates Following Previous Booster Vaccination or Breakthrough Infection
Source: Clin Infect Dis. 2024 Nov 26;80(2):316–22. doi: 10.1093/cid/ciae559 (PMC11848277; doi:10.1093/cid/ciae559)
Supplement: ciae559_Supplementary_Data [file ciae559_supplementary_data.zip › TableS2_southkorea.pdf]

Supplementary Table S2 South Korea

Infection date	Following year optimal booster date
19-Sep	19-Sep
20-Sep	19-Sep
21-Sep	19-Sep
22-Sep	19-Sep
23-Sep	19-Sep
24-Sep	19-Sep
25-Sep	20-Sep
26-Sep	20-Sep
27-Sep	20-Sep
28-Sep	20-Sep
29-Sep	20-Sep
30-Sep	20-Sep
1-Oct	20-Sep
2-Oct	20-Sep
3-Oct	20-Sep
4-Oct	20-Sep
5-Oct	20-Sep
6-Oct	20-Sep
7-Oct	20-Sep
8-Oct	20-Sep
9-Oct	20-Sep
10-Oct	20-Sep
11-Oct	20-Sep
12-Oct	20-Sep
13-Oct	20-Sep
14-Oct	20-Sep
15-Oct	20-Sep
16-Oct	20-Sep
17-Oct	20-Sep
18-Oct	20-Sep
19-Oct	20-Sep
20-Oct	20-Sep
21-Oct	20-Sep
22-Oct	20-Sep
23-Oct	20-Sep
24-Oct	21-Sep
25-Oct	21-Sep
26-Oct	21-Sep
27-Oct	21-Sep
28-Oct	21-Sep
29-Oct	21-Sep

30-Oct	21-Sep
31-Oct	21-Sep
1-Nov	21-Sep
2-Nov	21-Sep
3-Nov	21-Sep
4-Nov	21-Sep
5-Nov	21-Sep
6-Nov	21-Sep
7-Nov	21-Sep
8-Nov	21-Sep
9-Nov	21-Sep
10-Nov	21-Sep
11-Nov	21-Sep
12-Nov	21-Sep
13-Nov	21-Sep
14-Nov	21-Sep
15-Nov	21-Sep
16-Nov	21-Sep
17-Nov	21-Sep
18-Nov	21-Sep
19-Nov	21-Sep
20-Nov	21-Sep
21-Nov	21-Sep
22-Nov	21-Sep
23-Nov	21-Sep
24-Nov	21-Sep
25-Nov	21-Sep
26-Nov	22-Sep
27-Nov	22-Sep
28-Nov	22-Sep
29-Nov	22-Sep
30-Nov	22-Sep
1-Dec	22-Sep
2-Dec	22-Sep
3-Dec	22-Sep
4-Dec	22-Sep
5-Dec	22-Sep
6-Dec	22-Sep
7-Dec	22-Sep
8-Dec	22-Sep
9-Dec	22-Sep
10-Dec	22-Sep
11-Dec	22-Sep

12-Dec	22-Sep
13-Dec	22-Sep
14-Dec	22-Sep
15-Dec	22-Sep
16-Dec	22-Sep
17-Dec	22-Sep
18-Dec	22-Sep
19-Dec	22-Sep
20-Dec	22-Sep
21-Dec	22-Sep
22-Dec	22-Sep
23-Dec	22-Sep
24-Dec	22-Sep
25-Dec	22-Sep
26-Dec	22-Sep
27-Dec	22-Sep
28-Dec	22-Sep
29-Dec	22-Sep
30-Dec	22-Sep
31-Dec	22-Sep
1-Jan	22-Sep
2-Jan	22-Sep
3-Jan	22-Sep
4-Jan	22-Sep
5-Jan	22-Sep
6-Jan	23-Sep
7-Jan	23-Sep
8-Jan	23-Sep
9-Jan	23-Sep
10-Jan	23-Sep
11-Jan	23-Sep
12-Jan	23-Sep
13-Jan	23-Sep
14-Jan	23-Sep
15-Jan	23-Sep
16-Jan	23-Sep
17-Jan	23-Sep
18-Jan	23-Sep
19-Jan	23-Sep
20-Jan	10-Oct
21-Jan	10-Oct
22-Jan	10-Oct
23-Jan	10-Oct

24-Jan	10-Oct
25-Jan	10-Oct
26-Jan	10-Oct
27-Jan	10-Oct
28-Jan	11-Oct
29-Jan	11-Oct
30-Jan	11-Oct
31-Jan	11-Oct
1-Feb	11-Oct
2-Feb	11-Oct
3-Feb	11-Oct
4-Feb	11-Oct
5-Feb	12-Oct
6-Feb	12-Oct
7-Feb	12-Oct
8-Feb	12-Oct
9-Feb	12-Oct
10-Feb	12-Oct
11-Feb	12-Oct
12-Feb	12-Oct
13-Feb	13-Oct
14-Feb	13-Oct
15-Feb	13-Oct
16-Feb	13-Oct
17-Feb	13-Oct
18-Feb	13-Oct
19-Feb	13-Oct
20-Feb	13-Oct
21-Feb	13-Oct
22-Feb	14-Oct
23-Feb	14-Oct
24-Feb	14-Oct
25-Feb	14-Oct
26-Feb	14-Oct
27-Feb	14-Oct
28-Feb	14-Oct
1-Mar	14-Oct
2-Mar	14-Oct
3-Mar	14-Oct
4-Mar	15-Oct
5-Mar	15-Oct
6-Mar	15-Oct
7-Mar	15-Oct

8-Mar	15-Oct
9-Mar	15-Oct
10-Mar	15-Oct
11-Mar	15-Oct
12-Mar	15-Oct
13-Mar	15-Oct
14-Mar	16-Oct
15-Mar	16-Oct
16-Mar	16-Oct
17-Mar	16-Oct
18-Mar	16-Oct
19-Mar	16-Oct
20-Mar	16-Oct
21-Mar	16-Oct
22-Mar	16-Oct
23-Mar	16-Oct
24-Mar	17-Oct
25-Mar	17-Oct
26-Mar	17-Oct
27-Mar	17-Oct
28-Mar	17-Oct
29-Mar	17-Oct
30-Mar	17-Oct
31-Mar	17-Oct
1-Apr	17-Oct
2-Apr	17-Oct
3-Apr	17-Oct
4-Apr	17-Oct
5-Apr	17-Oct
6-Apr	17-Oct
7-Apr	17-Oct
8-Apr	17-Oct
9-Apr	17-Oct
10-Apr	17-Oct
11-Apr	17-Oct
12-Apr	17-Oct
13-Apr	17-Oct
14-Apr	17-Oct
15-Apr	17-Oct
16-Apr	17-Oct
17-Apr	17-Oct
18-Apr	17-Oct
19-Apr	17-Oct

20-Apr	17-Oct
21-Apr	17-Oct
22-Apr	17-Oct
23-Apr	17-Oct
24-Apr	17-Oct
25-Apr	17-Oct
26-Apr	17-Oct
27-Apr	17-Oct
28-Apr	17-Oct
29-Apr	17-Oct
30-Apr	17-Oct
1-May	17-Oct
2-May	17-Oct
3-May	17-Oct
4-May	17-Oct
5-May	17-Oct
6-May	17-Oct
7-May	17-Oct
8-May	17-Oct
9-May	17-Oct
10-May	17-Oct
11-May	17-Oct
12-May	17-Oct
13-May	17-Oct
14-May	17-Oct
15-May	17-Oct
16-May	17-Oct
17-May	17-Oct
18-May	17-Oct
19-May	17-Oct
20-May	17-Oct
21-May	17-Oct
22-May	17-Oct
23-May	17-Oct
24-May	17-Oct
25-May	17-Oct
26-May	17-Oct
27-May	17-Oct
28-May	17-Oct
29-May	17-Oct
30-May	17-Oct
31-May	17-Oct
1-Jun	17-Oct

2-Jun	17-Oct
3-Jun	17-Oct
4-Jun	18-Oct
5-Jun	18-Oct
6-Jun	18-Oct
7-Jun	18-Oct
8-Jun	18-Oct
9-Jun	18-Oct
10-Jun	18-Oct
11-Jun	18-Oct
12-Jun	18-Oct
13-Jun	18-Oct
14-Jun	18-Oct
15-Jun	18-Oct
16-Jun	18-Oct
17-Jun	18-Oct
18-Jun	18-Oct
19-Jun	18-Oct
20-Jun	18-Oct
21-Jun	18-Oct
22-Jun	18-Oct
23-Jun	18-Oct
24-Jun	18-Oct
25-Jun	18-Oct
26-Jun	18-Oct
27-Jun	18-Oct
28-Jun	18-Oct
29-Jun	18-Oct
30-Jun	18-Oct
1-Jul	18-Oct
2-Jul	18-Oct
3-Jul	18-Oct
4-Jul	18-Oct
5-Jul	18-Oct
6-Jul	18-Oct
7-Jul	18-Oct
8-Jul	18-Oct
9-Jul	18-Oct
10-Jul	18-Oct
11-Jul	18-Oct
12-Jul	18-Oct
13-Jul	18-Oct
14-Jul	18-Oct

15-Jul	18-Oct
16-Jul	18-Oct
17-Jul	18-Oct
18-Jul	18-Oct
19-Jul	18-Oct
20-Jul	31-Jan
21-Jul	28-Feb
22-Jul	28-Feb
23-Jul	28-Feb
24-Jul	28-Feb
25-Jul	28-Feb
26-Jul	28-Feb
27-Jul	28-Feb
28-Jul	28-Feb
29-Jul	28-Feb
30-Jul	28-Feb
31-Jul	28-Feb
1-Aug	28-Feb
2-Aug	28-Feb
3-Aug	28-Feb
4-Aug	28-Feb
5-Aug	28-Feb
6-Aug	28-Feb
7-Aug	28-Feb
8-Aug	28-Feb
9-Aug	28-Feb
10-Aug	28-Feb
11-Aug	28-Feb
12-Aug	28-Feb
13-Aug	28-Feb
14-Aug	28-Feb
15-Aug	28-Feb
16-Aug	28-Feb
17-Aug	28-Feb
18-Aug	28-Feb
19-Aug	28-Feb
20-Aug	28-Feb
21-Aug	28-Feb
22-Aug	28-Feb
23-Aug	28-Feb
24-Aug	28-Feb
25-Aug	28-Feb
26-Aug	28-Feb

27-Aug	28-Feb
28-Aug	28-Feb
29-Aug	28-Feb
30-Aug	28-Feb
31-Aug	28-Feb
1-Sep	28-Feb
2-Sep	28-Feb
3-Sep	28-Feb
4-Sep	28-Feb
5-Sep	28-Feb
6-Sep	28-Feb
7-Sep	28-Feb
8-Sep	28-Feb
9-Sep	28-Feb
10-Sep	28-Feb
11-Sep	28-Feb
12-Sep	28-Feb
13-Sep	28-Feb
14-Sep	28-Feb
15-Sep	28-Feb
16-Sep	28-Feb
17-Sep	28-Feb
18-Sep	28-Feb
