# Supplementary material for: Optimal Annual COVID-19 Vaccine Boosting Dates Following Previous Booster Vaccination or Breakthrough Infection
Source: Clin Infect Dis. 2024 Nov 26;80(2):316–22. doi: 10.1093/cid/ciae559 (PMC11848277; doi:10.1093/cid/ciae559)
Supplement: ciae559_Supplementary_Data [file ciae559_supplementary_data.zip › TableS3_equations2.pdf]

Table S3. Phenomenological equations for Figure 3

Location	Equation for yearly optimal booster vaccination date
New York	$25.80 - 8.08x + 1.31x^2 - 0.09x^3 + 3.73 \times 10^{-3}x^4 - 9.15 \times 10^{-5}x^5 + 1.49 \times 10^{-6}x^6 - 1.67 \times 10^{-8}x^7 + 1.33 \times 10^{-10}x^8 - 7.59 \times 10^{-13}x^9 + 3.13 \times 10^{-15}x^{10} - 9.21 \times 10^{-18}x^{11} + 1.89 \times 10^{-20}x^{12} - 2.56 \times 10^{-23}x^{13} + 2.06 \times 10^{-26}x^{14} - 7.43 \times 10^{-30}x^{15}$
Stockholm	$3.79 + 2.57x - 0.10x^2 + 1.62 \times 10^{-3}x^3 - 1.28 \times 10^{-5}x^4 + 5.31 \times 10^{-8}x^5 - 1.11 \times 10^{-10}x^6 + 9.20 \times 10^{-14}x^7$
South Korea	$33.17 - 2.60x + 0.11x^2 - 1.91 \times 10^{-3}x^3 + 1.60 \times 10^{-5}x^4 - 6.82 \times 10^{-8}x^5 + 1.43 \times 10^{-10}x^6 - 1.17 \times 10^{-13}x^7$
Edinburgh	$-2.56 + 1.66x - 0.06x^2 + 1.13 \times 10^{-3}x^3 - 9.62 \times 10^{-6}x^4 + 4.19 \times 10^{-8}x^5 - 8.88 \times 10^{-11}x^6 + 7.28 \times 10^{-14}x^7$
Yamagata	$1.49 + 1.83x - 0.06x^2 + 9.16 \times 10^{-3}x^3 - 6.20 \times 10^{-6}x^4 + 2.16 \times 10^{-8}x^5 - 3.79 \times 10^{-11}x^6 + 2.64 \times 10^{-14}x^7$
Nepal	$3.64 + 0.14x + 0.01x^2 - 2.15 \times 10^{-3}x^3 + 1.94 \times 10^{-6}x^4 - 8.50 \times 10^{-9}x^5 + 1.78 \times 10^{-11}x^6 - 1.42 \times 10^{-14}x^7$
Guangzhou	$13.82 - 3.91x + 0.62x^2 - 0.04x^3 + 1.58 \times 10^{-3}x^4 - 3.61 \times 10^{-5}x^5 + 5.38 \times 10^{-7}x^6 - 5.46 \times 10^{-9}x^7 + 3.88 \times 10^{-11}x^8 - 1.93 \times 10^{-13}x^9 + 6.73 \times 10^{-16}x^{10} - 1.60 \times 10^{-18}x^{11} + 2.48 \times 10^{-21}x^{12} - 2.22 \times 10^{-24}x^{13} + 8.27 \times 10^{-28}x^{14} + 6.11 \times 10^{-32}x^{15}$
Netherlands	$15.30 - 1.02x + 0.05x^2 - 8.64 \times 10^{-3}x^3 + 6.56 \times 10^{-6}x^4 - 2.56 \times 10^{-8}x^5 + 4.98 \times 10^{-11}x^6 - 3.81 \times 10^{-14}x^7$
Gothenburg	$-11.61 + 3.87x - 0.15x^2 + 2.46 \times 10^{-5}x^3 - 1.95 \times 10^{-5}x^4 + 7.97 \times 10^{-8}x^5 - 1.60 \times 10^{-10}x^6 + 1.25 \times 10^{-13}x^7$
Norway	$13.82 - 3.91x + 0.62x^2 - 0.04x^3 + 1.58 \times 10^{-3}x^4 - 3.61 \times 10^{-5}x^5 + 5.38 \times 10^{-7}x^6 - 5.46 \times 10^{-9}x^7 + 3.88 \times 10^{-11}x^8 - 1.93 \times 10^{-13}x^9 + 6.73 \times 10^{-16}x^{10} - 1.60 \times 10^{-18}x^{11} + 2.48 \times 10^{-21}x^{12} - 2.22 \times 10^{-24}x^{13} + 8.27 \times 10^{-28}x^{14} + 6.11 \times 10^{-32}x^{15}$
Israel	$28.41 - 1.03x + 0.04x^2 - 5.18 \times 10^{-3}x^3 + 3.94 \times 10^{-6}x^4 - 1.48 \times 10^{-8}x^5 + 2.62 \times 10^{-11}x^6 - 1.75 \times 10^{-14}x^7$
