# Supplementary material for: Optimal Annual COVID-19 Vaccine Boosting Dates Following Previous Booster Vaccination or Breakthrough Infection
Source: Clin Infect Dis. 2024 Nov 26;80(2):316–22. doi: 10.1093/cid/ciae559 (PMC11848277; doi:10.1093/cid/ciae559)
Supplement: ciae559_Supplementary_Data [file ciae559_supplementary_data.zip › TableS4_newyork.pdf]

Supplementary Table S4 New York

Infection date	Following year optimal booster date
15-Sep	15-Sep
16-Sep	15-Sep
17-Sep	15-Sep
18-Sep	15-Sep
19-Sep	15-Sep
20-Sep	15-Sep
21-Sep	15-Sep
22-Sep	15-Sep
23-Sep	15-Sep
24-Sep	15-Sep
25-Sep	15-Sep
26-Sep	15-Sep
27-Sep	15-Sep
28-Sep	15-Sep
29-Sep	15-Sep
30-Sep	15-Sep
1-Oct	15-Sep
2-Oct	15-Sep
3-Oct	15-Sep
4-Oct	15-Sep
5-Oct	15-Sep
6-Oct	15-Sep
7-Oct	15-Sep
8-Oct	15-Sep
9-Oct	15-Sep
10-Oct	15-Sep
11-Oct	15-Sep
12-Oct	15-Sep
13-Oct	15-Sep
14-Oct	15-Sep
15-Oct	15-Sep
16-Oct	15-Sep
17-Oct	15-Sep
18-Oct	15-Sep
19-Oct	15-Sep
20-Oct	15-Sep
21-Oct	15-Sep
22-Oct	15-Sep
23-Oct	15-Sep
24-Oct	15-Sep
25-Oct	15-Sep

26-Oct	15-Sep
27-Oct	15-Sep
28-Oct	15-Sep
29-Oct	15-Sep
30-Oct	15-Sep
31-Oct	15-Sep
1-Nov	15-Sep
2-Nov	15-Sep
3-Nov	15-Sep
4-Nov	15-Sep
5-Nov	15-Sep
6-Nov	15-Sep
7-Nov	15-Sep
8-Nov	15-Sep
9-Nov	15-Sep
10-Nov	15-Sep
11-Nov	15-Sep
12-Nov	15-Sep
13-Nov	15-Sep
14-Nov	15-Sep
15-Nov	15-Sep
16-Nov	15-Sep
17-Nov	15-Sep
18-Nov	15-Sep
19-Nov	15-Sep
20-Nov	15-Sep
21-Nov	15-Sep
22-Nov	15-Sep
23-Nov	15-Sep
24-Nov	15-Sep
25-Nov	15-Sep
26-Nov	15-Sep
27-Nov	15-Sep
28-Nov	15-Sep
29-Nov	15-Sep
30-Nov	15-Sep
1-Dec	15-Sep
2-Dec	15-Sep
3-Dec	15-Sep
4-Dec	15-Sep
5-Dec	15-Sep
6-Dec	15-Sep
7-Dec	15-Sep

8-Dec	15-Sep
9-Dec	15-Sep
10-Dec	15-Sep
11-Dec	15-Sep
12-Dec	15-Sep
13-Dec	15-Sep
14-Dec	15-Sep
15-Dec	15-Sep
16-Dec	15-Sep
17-Dec	15-Sep
18-Dec	15-Sep
19-Dec	15-Sep
20-Dec	15-Sep
21-Dec	15-Sep
22-Dec	15-Sep
23-Dec	15-Sep
24-Dec	15-Sep
25-Dec	15-Sep
26-Dec	15-Sep
27-Dec	15-Sep
28-Dec	15-Sep
29-Dec	15-Sep
30-Dec	16-Sep
31-Dec	16-Sep
1-Jan	16-Sep
2-Jan	16-Sep
3-Jan	16-Sep
4-Jan	16-Sep
5-Jan	16-Sep
6-Jan	16-Sep
7-Jan	16-Sep
8-Jan	16-Sep
9-Jan	16-Sep
10-Jan	16-Sep
11-Jan	16-Sep
12-Jan	16-Sep
13-Jan	16-Sep
14-Jan	16-Sep
15-Jan	16-Sep
16-Jan	16-Sep
17-Jan	16-Sep
18-Jan	16-Sep
19-Jan	16-Sep

20-Jan	16-Sep
21-Jan	16-Sep
22-Jan	16-Sep
23-Jan	16-Sep
24-Jan	16-Sep
25-Jan	16-Sep
26-Jan	16-Sep
27-Jan	16-Sep
28-Jan	16-Sep
29-Jan	16-Sep
30-Jan	16-Sep
31-Jan	16-Sep
1-Feb	16-Sep
2-Feb	16-Sep
3-Feb	16-Sep
4-Feb	16-Sep
5-Feb	16-Sep
6-Feb	16-Sep
7-Feb	16-Sep
8-Feb	16-Sep
9-Feb	16-Sep
10-Feb	16-Sep
11-Feb	16-Sep
12-Feb	16-Sep
13-Feb	16-Sep
14-Feb	16-Sep
15-Feb	16-Sep
16-Feb	16-Sep
17-Feb	16-Sep
18-Feb	16-Sep
19-Feb	16-Sep
20-Feb	16-Sep
21-Feb	16-Sep
22-Feb	16-Sep
23-Feb	16-Sep
24-Feb	16-Sep
25-Feb	16-Sep
26-Feb	16-Sep
27-Feb	16-Sep
28-Feb	16-Sep
1-Mar	16-Sep
2-Mar	16-Sep
3-Mar	16-Sep

4-Mar	16-Sep
5-Mar	16-Sep
6-Mar	16-Sep
7-Mar	16-Sep
8-Mar	16-Sep
9-Mar	16-Sep
10-Mar	16-Sep
11-Mar	16-Sep
12-Mar	16-Sep
13-Mar	16-Sep
14-Mar	16-Sep
15-Mar	16-Sep
16-Mar	16-Sep
17-Mar	16-Sep
18-Mar	16-Sep
19-Mar	16-Sep
20-Mar	16-Sep
21-Mar	16-Sep
22-Mar	16-Sep
23-Mar	16-Sep
24-Mar	16-Sep
25-Mar	16-Sep
26-Mar	16-Sep
27-Mar	16-Sep
28-Mar	16-Sep
29-Mar	16-Sep
30-Mar	16-Sep
31-Mar	16-Sep
1-Apr	16-Sep
2-Apr	16-Sep
3-Apr	16-Sep
4-Apr	16-Sep
5-Apr	16-Sep
6-Apr	16-Sep
7-Apr	16-Sep
8-Apr	16-Sep
9-Apr	16-Sep
10-Apr	16-Sep
11-Apr	16-Sep
12-Apr	16-Sep
13-Apr	16-Sep
14-Apr	16-Sep
15-Apr	16-Sep

16-Apr	16-Sep
17-Apr	17-Sep
18-Apr	17-Sep
19-Apr	17-Sep
20-Apr	17-Sep
21-Apr	17-Sep
22-Apr	17-Sep
23-Apr	17-Sep
24-Apr	17-Sep
25-Apr	17-Sep
26-Apr	17-Sep
27-Apr	17-Sep
28-Apr	17-Sep
29-Apr	17-Sep
30-Apr	17-Sep
1-May	17-Sep
2-May	17-Sep
3-May	17-Sep
4-May	17-Sep
5-May	17-Sep
6-May	17-Sep
7-May	17-Sep
8-May	17-Sep
9-May	17-Sep
10-May	28-Nov
11-May	28-Nov
12-May	28-Nov
13-May	28-Nov
14-May	28-Nov
15-May	28-Nov
16-May	28-Nov
17-May	28-Nov
18-May	28-Nov
19-May	28-Nov
20-May	28-Nov
21-May	28-Nov
22-May	28-Nov
23-May	28-Nov
24-May	28-Nov
25-May	28-Nov
26-May	28-Nov
27-May	28-Nov
28-May	28-Nov

29-May	28-Nov
30-May	28-Nov
31-May	28-Nov
1-Jun	28-Nov
2-Jun	28-Nov
3-Jun	29-Nov
4-Jun	29-Nov
5-Jun	30-Nov
6-Jun	1-Dec
7-Jun	1-Dec
8-Jun	2-Dec
9-Jun	2-Dec
10-Jun	3-Dec
11-Jun	3-Dec
12-Jun	4-Dec
13-Jun	5-Dec
14-Jun	5-Dec
15-Jun	5-Dec
16-Jun	5-Dec
17-Jun	6-Dec
18-Jun	6-Dec
19-Jun	6-Dec
20-Jun	6-Dec
21-Jun	7-Dec
22-Jun	7-Dec
23-Jun	7-Dec
24-Jun	7-Dec
25-Jun	8-Dec
26-Jun	8-Dec
27-Jun	8-Dec
28-Jun	8-Dec
29-Jun	9-Dec
30-Jun	9-Dec
1-Jul	9-Dec
2-Jul	9-Dec
3-Jul	10-Dec
4-Jul	10-Dec
5-Jul	10-Dec
6-Jul	10-Dec
7-Jul	11-Dec
8-Jul	11-Dec
9-Jul	11-Dec
10-Jul	11-Dec

11-Jul	29-Jan
12-Jul	29-Jan
13-Jul	29-Jan
14-Jul	29-Jan
15-Jul	29-Jan
16-Jul	29-Jan
17-Jul	29-Jan
18-Jul	29-Jan
19-Jul	29-Jan
20-Jul	29-Jan
21-Jul	29-Jan
22-Jul	29-Jan
23-Jul	29-Jan
24-Jul	29-Jan
25-Jul	29-Jan
26-Jul	29-Jan
27-Jul	29-Jan
28-Jul	29-Jan
29-Jul	29-Jan
30-Jul	29-Jan
31-Jul	29-Jan
1-Aug	29-Jan
2-Aug	29-Jan
3-Aug	29-Jan
4-Aug	29-Jan
5-Aug	26-Feb
6-Aug	26-Feb
7-Aug	26-Feb
8-Aug	26-Feb
9-Aug	26-Feb
10-Aug	26-Feb
11-Aug	26-Feb
12-Aug	26-Feb
13-Aug	26-Feb
14-Aug	26-Feb
15-Aug	26-Feb
16-Aug	26-Feb
17-Aug	26-Feb
18-Aug	26-Feb
19-Aug	26-Feb
20-Aug	26-Feb
21-Aug	26-Feb
22-Aug	26-Feb

23-Aug	26-Feb
24-Aug	26-Feb
25-Aug	26-Feb
26-Aug	26-Feb
27-Aug	26-Feb
28-Aug	26-Feb
29-Aug	26-Feb
30-Aug	29-Mar
31-Aug	29-Mar
1-Sep	29-Mar
2-Sep	29-Mar
3-Sep	29-Mar
4-Sep	29-Mar
5-Sep	29-Mar
6-Sep	29-Mar
7-Sep	29-Mar
8-Sep	29-Mar
9-Sep	29-Mar
10-Sep	29-Mar
11-Sep	29-Mar
12-Sep	29-Mar
13-Sep	29-Mar
14-Sep	29-Mar
