# Supplementary material for: Optimal Annual COVID-19 Vaccine Boosting Dates Following Previous Booster Vaccination or Breakthrough Infection
Source: Clin Infect Dis. 2024 Nov 26;80(2):316–22. doi: 10.1093/cid/ciae559 (PMC11848277; doi:10.1093/cid/ciae559)
Supplement: ciae559_Supplementary_Data [file ciae559_supplementary_data.zip › TableS5_yamagata.pdf]

Supplementary Table S5	Yamagata
**Infection date**	**Following year optimal booster date**
18-Oct	18-Oct
19-Oct	18-Oct
20-Oct	18-Oct
21-Oct	18-Oct
22-Oct	18-Oct
23-Oct	18-Oct
24-Oct	18-Oct
25-Oct	18-Oct
26-Oct	18-Oct
27-Oct	19-Oct
28-Oct	19-Oct
29-Oct	19-Oct
30-Oct	19-Oct
31-Oct	19-Oct
1-Nov	19-Oct
2-Nov	19-Oct
3-Nov	19-Oct
4-Nov	19-Oct
5-Nov	19-Oct
6-Nov	20-Oct
7-Nov	20-Oct
8-Nov	20-Oct
9-Nov	20-Oct
10-Nov	20-Oct
11-Nov	20-Oct
12-Nov	20-Oct
13-Nov	20-Oct
14-Nov	20-Oct
15-Nov	20-Oct
16-Nov	21-Oct
17-Nov	21-Oct
18-Nov	21-Oct
19-Nov	21-Oct
20-Nov	21-Oct
21-Nov	21-Oct
22-Nov	21-Oct
23-Nov	21-Oct
24-Nov	21-Oct
25-Nov	21-Oct
26-Nov	21-Oct
27-Nov	22-Oct

28-Nov	22-Oct
29-Nov	22-Oct
30-Nov	22-Oct
1-Dec	22-Oct
2-Dec	22-Oct
3-Dec	22-Oct
4-Dec	22-Oct
5-Dec	22-Oct
6-Dec	22-Oct
7-Dec	22-Oct
8-Dec	23-Oct
9-Dec	23-Oct
10-Dec	23-Oct
11-Dec	23-Oct
12-Dec	23-Oct
13-Dec	23-Oct
14-Dec	23-Oct
15-Dec	23-Oct
16-Dec	23-Oct
17-Dec	23-Oct
18-Dec	23-Oct
19-Dec	23-Oct
20-Dec	24-Oct
21-Dec	24-Oct
22-Dec	24-Oct
23-Dec	24-Oct
24-Dec	24-Oct
25-Dec	24-Oct
26-Dec	24-Oct
27-Dec	24-Oct
28-Dec	24-Oct
29-Dec	25-Oct
30-Dec	25-Oct
31-Dec	25-Oct
1-Jan	25-Oct
2-Jan	25-Oct
3-Jan	25-Oct
4-Jan	25-Oct
5-Jan	25-Oct
6-Jan	25-Oct
7-Jan	25-Oct
8-Jan	26-Oct
9-Jan	26-Oct

10-Jan	26-Oct
11-Jan	26-Oct
12-Jan	26-Oct
13-Jan	26-Oct
14-Jan	26-Oct
15-Jan	26-Oct
16-Jan	26-Oct
17-Jan	27-Oct
18-Jan	8-Nov
19-Jan	9-Nov
20-Jan	9-Nov
21-Jan	10-Nov
22-Jan	11-Nov
23-Jan	11-Nov
24-Jan	30-Nov
25-Jan	30-Nov
26-Jan	30-Nov
27-Jan	30-Nov
28-Jan	30-Nov
29-Jan	30-Nov
30-Jan	30-Nov
31-Jan	30-Nov
1-Feb	30-Nov
2-Feb	30-Nov
3-Feb	30-Nov
4-Feb	30-Nov
5-Feb	30-Nov
6-Feb	30-Nov
7-Feb	30-Nov
8-Feb	30-Nov
9-Feb	30-Nov
10-Feb	30-Nov
11-Feb	1-Dec
12-Feb	1-Dec
13-Feb	1-Dec
14-Feb	2-Dec
15-Feb	2-Dec
16-Feb	3-Dec
17-Feb	3-Dec
18-Feb	4-Dec
19-Feb	4-Dec
20-Feb	4-Dec
21-Feb	5-Dec

22-Feb	5-Dec
23-Feb	6-Dec
24-Feb	6-Dec
25-Feb	7-Dec
26-Feb	7-Dec
27-Feb	7-Dec
28-Feb	7-Dec
1-Mar	7-Dec
2-Mar	7-Dec
3-Mar	7-Dec
4-Mar	7-Dec
5-Mar	8-Dec
6-Mar	8-Dec
7-Mar	8-Dec
8-Mar	8-Dec
9-Mar	8-Dec
10-Mar	8-Dec
11-Mar	8-Dec
12-Mar	9-Dec
13-Mar	9-Dec
14-Mar	9-Dec
15-Mar	9-Dec
16-Mar	9-Dec
17-Mar	9-Dec
18-Mar	9-Dec
19-Mar	9-Dec
20-Mar	10-Dec
21-Mar	10-Dec
22-Mar	10-Dec
23-Mar	10-Dec
24-Mar	10-Dec
25-Mar	10-Dec
26-Mar	10-Dec
27-Mar	10-Dec
28-Mar	11-Dec
29-Mar	11-Dec
30-Mar	11-Dec
31-Mar	11-Dec
1-Apr	11-Dec
2-Apr	11-Dec
3-Apr	11-Dec
4-Apr	11-Dec
5-Apr	11-Dec

6-Apr	12-Dec
7-Apr	12-Dec
8-Apr	12-Dec
9-Apr	12-Dec
10-Apr	12-Dec
11-Apr	12-Dec
12-Apr	12-Dec
13-Apr	12-Dec
14-Apr	12-Dec
15-Apr	13-Dec
16-Apr	13-Dec
17-Apr	13-Dec
18-Apr	13-Dec
19-Apr	13-Dec
20-Apr	13-Dec
21-Apr	13-Dec
22-Apr	13-Dec
23-Apr	13-Dec
24-Apr	14-Dec
25-Apr	14-Dec
26-Apr	14-Dec
27-Apr	14-Dec
28-Apr	14-Dec
29-Apr	14-Dec
30-Apr	14-Dec
1-May	14-Dec
2-May	14-Dec
3-May	14-Dec
4-May	15-Dec
5-May	15-Dec
6-May	15-Dec
7-May	15-Dec
8-May	15-Dec
9-May	15-Dec
10-May	15-Dec
11-May	15-Dec
12-May	15-Dec
13-May	15-Dec
14-May	15-Dec
15-May	16-Dec
16-May	16-Dec
17-May	16-Dec
18-May	16-Dec

19-May	16-Dec
20-May	16-Dec
21-May	16-Dec
22-May	16-Dec
23-May	16-Dec
24-May	16-Dec
25-May	16-Dec
26-May	17-Dec
27-May	17-Dec
28-May	17-Dec
29-May	17-Dec
30-May	17-Dec
31-May	17-Dec
1-Jun	17-Dec
2-Jun	17-Dec
3-Jun	17-Dec
4-Jun	17-Dec
5-Jun	17-Dec
6-Jun	17-Dec
7-Jun	17-Dec
8-Jun	17-Dec
9-Jun	17-Dec
10-Jun	17-Dec
11-Jun	17-Dec
12-Jun	17-Dec
13-Jun	17-Dec
14-Jun	17-Dec
15-Jun	17-Dec
16-Jun	17-Dec
17-Jun	17-Dec
18-Jun	17-Dec
19-Jun	17-Dec
20-Jun	17-Dec
21-Jun	17-Dec
22-Jun	17-Dec
23-Jun	17-Dec
24-Jun	17-Dec
25-Jun	17-Dec
26-Jun	17-Dec
27-Jun	17-Dec
28-Jun	17-Dec
29-Jun	17-Dec
30-Jun	17-Dec

1-Jul	17-Dec
2-Jul	17-Dec
3-Jul	17-Dec
4-Jul	17-Dec
5-Jul	17-Dec
6-Jul	17-Dec
7-Jul	17-Dec
8-Jul	17-Dec
9-Jul	17-Dec
10-Jul	17-Dec
11-Jul	17-Dec
12-Jul	17-Dec
13-Jul	17-Dec
14-Jul	17-Dec
15-Jul	17-Dec
16-Jul	17-Dec
17-Jul	17-Dec
18-Jul	17-Dec
19-Jul	17-Dec
20-Jul	17-Dec
21-Jul	17-Dec
22-Jul	17-Dec
23-Jul	17-Dec
24-Jul	17-Dec
25-Jul	17-Dec
26-Jul	17-Dec
27-Jul	17-Dec
28-Jul	17-Dec
29-Jul	17-Dec
30-Jul	17-Dec
31-Jul	17-Dec
1-Aug	17-Dec
2-Aug	17-Dec
3-Aug	17-Dec
4-Aug	17-Dec
5-Aug	17-Dec
6-Aug	17-Dec
7-Aug	17-Dec
8-Aug	17-Dec
9-Aug	17-Dec
10-Aug	17-Dec
11-Aug	17-Dec
12-Aug	17-Dec

13-Aug	17-Dec
14-Aug	17-Dec
15-Aug	17-Dec
16-Aug	17-Dec
17-Aug	17-Dec
18-Aug	18-Dec
19-Aug	18-Dec
20-Aug	18-Dec
21-Aug	18-Dec
22-Aug	18-Dec
23-Aug	18-Dec
24-Aug	18-Dec
25-Aug	18-Dec
26-Aug	18-Dec
27-Aug	18-Dec
28-Aug	18-Dec
29-Aug	18-Dec
30-Aug	18-Dec
31-Aug	18-Dec
1-Sep	18-Dec
2-Sep	18-Dec
3-Sep	18-Dec
4-Sep	18-Dec
5-Sep	18-Dec
6-Sep	18-Dec
7-Sep	18-Dec
8-Sep	18-Dec
9-Sep	18-Dec
10-Sep	18-Dec
11-Sep	18-Dec
12-Sep	18-Dec
13-Sep	18-Dec
14-Sep	18-Dec
15-Sep	18-Dec
16-Sep	18-Dec
17-Sep	18-Dec
18-Sep	18-Dec
19-Sep	18-Dec
20-Sep	18-Dec
21-Sep	18-Dec
22-Sep	18-Dec
23-Sep	18-Dec
24-Sep	18-Dec

25-Sep	18-Dec
26-Sep	18-Dec
27-Sep	31-Mar
28-Sep	31-Mar
29-Sep	31-Mar
30-Sep	31-Mar
1-Oct	31-Mar
2-Oct	31-Mar
3-Oct	31-Mar
4-Oct	31-Mar
5-Oct	31-Mar
6-Oct	31-May
7-Oct	31-May
8-Oct	31-May
9-Oct	31-May
10-Oct	31-May
11-Oct	31-May
12-Oct	31-May
13-Oct	31-May
14-Oct	31-May
15-Oct	31-May
16-Oct	31-May
17-Oct	31-May
