# Supplementary material for: Optimal Annual COVID-19 Vaccine Boosting Dates Following Previous Booster Vaccination or Breakthrough Infection
Source: Clin Infect Dis. 2024 Nov 26;80(2):316–22. doi: 10.1093/cid/ciae559 (PMC11848277; doi:10.1093/cid/ciae559)
Supplement: ciae559_Supplementary_Data [file ciae559_supplementary_data.zip › TableS6_nepal.pdf]

Supplementary Table S6	Nepal
**Infection date**	**Following year optimal booster date**
11-Sep	11-Sep
12-Sep	11-Sep
13-Sep	11-Sep
14-Sep	12-Sep
15-Sep	12-Sep
16-Sep	13-Sep
17-Sep	13-Sep
18-Sep	14-Sep
19-Sep	14-Sep
20-Sep	14-Sep
21-Sep	15-Sep
22-Sep	15-Sep
23-Sep	16-Sep
24-Sep	16-Sep
25-Sep	16-Sep
26-Sep	16-Sep
27-Sep	17-Sep
28-Sep	17-Sep
29-Sep	17-Sep
30-Sep	17-Sep
1-Oct	18-Sep
2-Oct	18-Sep
3-Oct	18-Sep
4-Oct	19-Sep
5-Oct	19-Sep
6-Oct	19-Sep
7-Oct	19-Sep
8-Oct	20-Sep
9-Oct	20-Sep
10-Oct	20-Sep
11-Oct	21-Sep
12-Oct	21-Sep
13-Oct	21-Sep
14-Oct	21-Sep
15-Oct	22-Sep
16-Oct	22-Sep
17-Oct	22-Sep
18-Oct	22-Sep
19-Oct	23-Sep
20-Oct	23-Sep
21-Oct	23-Sep

22-Oct	24-Sep
23-Oct	24-Sep
24-Oct	24-Sep
25-Oct	25-Sep
26-Oct	25-Sep
27-Oct	25-Sep
28-Oct	26-Sep
29-Oct	26-Sep
30-Oct	26-Sep
31-Oct	26-Sep
1-Nov	27-Sep
2-Nov	27-Sep
3-Nov	27-Sep
4-Nov	28-Sep
5-Nov	28-Sep
6-Nov	28-Sep
7-Nov	29-Sep
8-Nov	29-Sep
9-Nov	2-Oct
10-Nov	2-Oct
11-Nov	2-Oct
12-Nov	3-Oct
13-Nov	3-Oct
14-Nov	4-Oct
15-Nov	4-Oct
16-Nov	5-Oct
17-Nov	5-Oct
18-Nov	6-Oct
19-Nov	6-Oct
20-Nov	7-Oct
21-Nov	7-Oct
22-Nov	8-Oct
23-Nov	8-Oct
24-Nov	8-Oct
25-Nov	9-Oct
26-Nov	9-Oct
27-Nov	10-Oct
28-Nov	10-Oct
29-Nov	11-Oct
30-Nov	11-Oct
1-Dec	11-Oct
2-Dec	12-Oct
3-Dec	12-Oct

4-Dec	13-Oct
5-Dec	13-Oct
6-Dec	14-Oct
7-Dec	14-Oct
8-Dec	14-Oct
9-Dec	15-Oct
10-Dec	15-Oct
11-Dec	16-Oct
12-Dec	16-Oct
13-Dec	17-Oct
14-Dec	17-Oct
15-Dec	17-Oct
16-Dec	17-Oct
17-Dec	18-Oct
18-Dec	18-Oct
19-Dec	18-Oct
20-Dec	19-Oct
21-Dec	19-Oct
22-Dec	19-Oct
23-Dec	19-Oct
24-Dec	20-Oct
25-Dec	20-Oct
26-Dec	20-Oct
27-Dec	20-Oct
28-Dec	21-Oct
29-Dec	21-Oct
30-Dec	21-Oct
31-Dec	22-Oct
1-Jan	22-Oct
2-Jan	22-Oct
3-Jan	22-Oct
4-Jan	23-Oct
5-Jan	23-Oct
6-Jan	23-Oct
7-Jan	24-Oct
8-Jan	24-Oct
9-Jan	24-Oct
10-Jan	24-Oct
11-Jan	25-Oct
12-Jan	25-Oct
13-Jan	25-Oct
14-Jan	26-Oct
15-Jan	26-Oct

16-Jan	26-Oct
17-Jan	27-Oct
18-Jan	27-Oct
19-Jan	27-Oct
20-Jan	28-Oct
21-Jan	28-Oct
22-Jan	28-Oct
23-Jan	29-Oct
24-Jan	29-Oct
25-Jan	2-Nov
26-Jan	3-Nov
27-Jan	3-Nov
28-Jan	4-Nov
29-Jan	4-Nov
30-Jan	5-Nov
31-Jan	5-Nov
1-Feb	6-Nov
2-Feb	6-Nov
3-Feb	7-Nov
4-Feb	7-Nov
5-Feb	8-Nov
6-Feb	9-Nov
7-Feb	9-Nov
8-Feb	10-Nov
9-Feb	10-Nov
10-Feb	11-Nov
11-Feb	11-Nov
12-Feb	12-Nov
13-Feb	12-Nov
14-Feb	13-Nov
15-Feb	14-Nov
16-Feb	14-Nov
17-Feb	15-Nov
18-Feb	15-Nov
19-Feb	17-Nov
20-Feb	19-Nov
21-Feb	21-Nov
22-Feb	23-Nov
23-Feb	24-Nov
24-Feb	25-Nov
25-Feb	26-Nov
26-Feb	27-Nov
27-Feb	28-Nov

28-Feb	29-Nov
1-Mar	30-Nov
2-Mar	30-Nov
3-Mar	30-Nov
4-Mar	30-Nov
5-Mar	30-Nov
6-Mar	30-Nov
7-Mar	30-Nov
8-Mar	30-Nov
9-Mar	30-Nov
10-Mar	30-Nov
11-Mar	30-Nov
12-Mar	30-Nov
13-Mar	30-Nov
14-Mar	30-Nov
15-Mar	30-Nov
16-Mar	1-Dec
17-Mar	1-Dec
18-Mar	1-Dec
19-Mar	2-Dec
20-Mar	2-Dec
21-Mar	3-Dec
22-Mar	3-Dec
23-Mar	4-Dec
24-Mar	4-Dec
25-Mar	5-Dec
26-Mar	5-Dec
27-Mar	6-Dec
28-Mar	6-Dec
29-Mar	7-Dec
30-Mar	7-Dec
31-Mar	7-Dec
1-Apr	7-Dec
2-Apr	7-Dec
3-Apr	7-Dec
4-Apr	8-Dec
5-Apr	8-Dec
6-Apr	8-Dec
7-Apr	8-Dec
8-Apr	8-Dec
9-Apr	8-Dec
10-Apr	9-Dec
11-Apr	9-Dec

12-Apr	9-Dec
13-Apr	9-Dec
14-Apr	9-Dec
15-Apr	10-Dec
16-Apr	10-Dec
17-Apr	10-Dec
18-Apr	10-Dec
19-Apr	10-Dec
20-Apr	10-Dec
21-Apr	11-Dec
22-Apr	11-Dec
23-Apr	11-Dec
24-Apr	11-Dec
25-Apr	11-Dec
26-Apr	11-Dec
27-Apr	11-Dec
28-Apr	12-Dec
29-Apr	12-Dec
30-Apr	12-Dec
1-May	12-Dec
2-May	12-Dec
3-May	12-Dec
4-May	13-Dec
5-May	13-Dec
6-May	13-Dec
7-May	13-Dec
8-May	13-Dec
9-May	13-Dec
10-May	13-Dec
11-May	14-Dec
12-May	14-Dec
13-May	14-Dec
14-May	14-Dec
15-May	14-Dec
16-May	14-Dec
17-May	14-Dec
18-May	15-Dec
19-May	15-Dec
20-May	15-Dec
21-May	15-Dec
22-May	15-Dec
23-May	15-Dec
24-May	15-Dec

25-May	16-Dec
26-May	16-Dec
27-May	16-Dec
28-May	16-Dec
29-May	16-Dec
30-May	16-Dec
31-May	16-Dec
1-Jun	17-Dec
2-Jun	17-Dec
3-Jun	17-Dec
4-Jun	17-Dec
5-Jun	17-Dec
6-Jun	17-Dec
7-Jun	17-Dec
8-Jun	17-Dec
9-Jun	17-Dec
10-Jun	17-Dec
11-Jun	17-Dec
12-Jun	17-Dec
13-Jun	17-Dec
14-Jun	17-Dec
15-Jun	17-Dec
16-Jun	17-Dec
17-Jun	17-Dec
18-Jun	17-Dec
19-Jun	17-Dec
20-Jun	17-Dec
21-Jun	17-Dec
22-Jun	17-Dec
23-Jun	17-Dec
24-Jun	17-Dec
25-Jun	17-Dec
26-Jun	17-Dec
27-Jun	17-Dec
28-Jun	17-Dec
29-Jun	17-Dec
30-Jun	17-Dec
1-Jul	17-Dec
2-Jul	17-Dec
3-Jul	17-Dec
4-Jul	17-Dec
5-Jul	17-Dec
6-Jul	17-Dec

7-Jul	17-Dec
8-Jul	17-Dec
9-Jul	17-Dec
10-Jul	17-Dec
11-Jul	18-Dec
12-Jul	18-Dec
13-Jul	18-Dec
14-Jul	18-Dec
15-Jul	18-Dec
16-Jul	18-Dec
17-Jul	18-Dec
18-Jul	18-Dec
19-Jul	18-Dec
20-Jul	18-Dec
21-Jul	18-Dec
22-Jul	18-Dec
23-Jul	18-Dec
24-Jul	18-Dec
25-Jul	18-Dec
26-Jul	18-Dec
27-Jul	18-Dec
28-Jul	18-Dec
29-Jul	18-Dec
30-Jul	18-Dec
31-Jul	18-Dec
1-Aug	18-Dec
2-Aug	18-Dec
3-Aug	18-Dec
4-Aug	18-Dec
5-Aug	18-Dec
6-Aug	18-Dec
7-Aug	18-Dec
8-Aug	18-Dec
9-Aug	18-Dec
10-Aug	18-Dec
11-Aug	18-Dec
12-Aug	18-Dec
13-Aug	18-Dec
14-Aug	18-Dec
15-Aug	18-Dec
16-Aug	18-Dec
17-Aug	18-Dec
18-Aug	18-Dec

19-Aug	18-Dec
20-Aug	18-Dec
21-Aug	18-Dec
22-Aug	18-Dec
23-Aug	18-Dec
24-Aug	18-Dec
25-Aug	18-Dec
26-Aug	18-Dec
27-Aug	31-Mar
28-Aug	31-Mar
29-Aug	31-Mar
30-Aug	31-Mar
31-Aug	31-Mar
1-Sep	31-Mar
2-Sep	31-Mar
3-Sep	31-Mar
4-Sep	31-Mar
5-Sep	31-Mar
6-Sep	31-Mar
7-Sep	31-Mar
8-Sep	31-Mar
9-Sep	31-Mar
10-Sep	31-Mar
