# Supplementary material for: Optimal Annual COVID-19 Vaccine Boosting Dates Following Previous Booster Vaccination or Breakthrough Infection
Source: Clin Infect Dis. 2024 Nov 26;80(2):316–22. doi: 10.1093/cid/ciae559 (PMC11848277; doi:10.1093/cid/ciae559)
Supplement: ciae559_Supplementary_Data [file ciae559_supplementary_data.zip › TableS7_guangzhou.pdf]

Supplementary Table S7	Guangzhou
**Infection date**	**Following year optimal booster date**
10-Oct	10-Oct
11-Oct	10-Oct
12-Oct	10-Oct
13-Oct	10-Oct
14-Oct	10-Oct
15-Oct	10-Oct
16-Oct	11-Oct
17-Oct	11-Oct
18-Oct	11-Oct
19-Oct	11-Oct
20-Oct	11-Oct
21-Oct	11-Oct
22-Oct	11-Oct
23-Oct	12-Oct
24-Oct	12-Oct
25-Oct	12-Oct
26-Oct	12-Oct
27-Oct	12-Oct
28-Oct	12-Oct
29-Oct	13-Oct
30-Oct	13-Oct
31-Oct	13-Oct
1-Nov	13-Oct
2-Nov	13-Oct
3-Nov	13-Oct
4-Nov	13-Oct
5-Nov	14-Oct
6-Nov	14-Oct
7-Nov	14-Oct
8-Nov	14-Oct
9-Nov	14-Oct
10-Nov	14-Oct
11-Nov	14-Oct
12-Nov	15-Oct
13-Nov	15-Oct
14-Nov	15-Oct
15-Nov	15-Oct
16-Nov	15-Oct
17-Nov	15-Oct
18-Nov	15-Oct
19-Nov	16-Oct

20-Nov	16-Oct
21-Nov	16-Oct
22-Nov	16-Oct
23-Nov	16-Oct
24-Nov	16-Oct
25-Nov	16-Oct
26-Nov	17-Oct
27-Nov	17-Oct
28-Nov	17-Oct
29-Nov	17-Oct
30-Nov	17-Oct
1-Dec	17-Oct
2-Dec	17-Oct
3-Dec	17-Oct
4-Dec	17-Oct
5-Dec	17-Oct
6-Dec	17-Oct
7-Dec	17-Oct
8-Dec	17-Oct
9-Dec	17-Oct
10-Dec	17-Oct
11-Dec	17-Oct
12-Dec	17-Oct
13-Dec	17-Oct
14-Dec	17-Oct
15-Dec	17-Oct
16-Dec	17-Oct
17-Dec	17-Oct
18-Dec	17-Oct
19-Dec	17-Oct
20-Dec	17-Oct
21-Dec	17-Oct
22-Dec	17-Oct
23-Dec	17-Oct
24-Dec	17-Oct
25-Dec	17-Oct
26-Dec	17-Oct
27-Dec	18-Oct
28-Dec	18-Oct
29-Dec	18-Oct
30-Dec	18-Oct
31-Dec	18-Oct
1-Jan	18-Oct

2-Jan	18-Oct
3-Jan	18-Oct
4-Jan	18-Oct
5-Jan	18-Oct
6-Jan	18-Oct
7-Jan	18-Oct
8-Jan	18-Oct
9-Jan	18-Oct
10-Jan	18-Oct
11-Jan	18-Oct
12-Jan	18-Oct
13-Jan	18-Oct
14-Jan	18-Oct
15-Jan	18-Oct
16-Jan	18-Oct
17-Jan	18-Oct
18-Jan	18-Oct
19-Jan	18-Oct
20-Jan	18-Oct
21-Jan	18-Oct
22-Jan	18-Oct
23-Jan	18-Oct
24-Jan	18-Oct
25-Jan	18-Oct
26-Jan	18-Oct
27-Jan	18-Oct
28-Jan	18-Oct
29-Jan	18-Oct
30-Jan	18-Oct
31-Jan	18-Oct
1-Feb	18-Oct
2-Feb	18-Oct
3-Feb	19-Oct
4-Feb	19-Oct
5-Feb	19-Oct
6-Feb	19-Oct
7-Feb	19-Oct
8-Feb	19-Oct
9-Feb	19-Oct
10-Feb	19-Oct
11-Feb	19-Oct
12-Feb	19-Oct
13-Feb	19-Oct

14-Feb	19-Oct
15-Feb	19-Oct
16-Feb	19-Oct
17-Feb	19-Oct
18-Feb	19-Oct
19-Feb	19-Oct
20-Feb	19-Oct
21-Feb	19-Oct
22-Feb	19-Oct
23-Feb	19-Oct
24-Feb	19-Oct
25-Feb	19-Oct
26-Feb	19-Oct
27-Feb	19-Oct
28-Feb	19-Oct
1-Mar	19-Oct
2-Mar	19-Oct
3-Mar	19-Oct
4-Mar	19-Oct
5-Mar	19-Oct
6-Mar	19-Oct
7-Mar	19-Oct
8-Mar	19-Oct
9-Mar	19-Oct
10-Mar	19-Oct
11-Mar	19-Oct
12-Mar	19-Oct
13-Mar	19-Oct
14-Mar	19-Oct
15-Mar	19-Oct
16-Mar	19-Oct
17-Mar	19-Oct
18-Mar	19-Oct
19-Mar	19-Oct
20-Mar	19-Oct
21-Mar	19-Oct
22-Mar	19-Oct
23-Mar	19-Oct
24-Mar	19-Oct
25-Mar	20-Oct
26-Mar	20-Oct
27-Mar	20-Oct
28-Mar	20-Oct

29-Mar	20-Oct
30-Mar	20-Oct
31-Mar	20-Oct
1-Apr	20-Oct
2-Apr	20-Oct
3-Apr	20-Oct
4-Apr	20-Oct
5-Apr	20-Oct
6-Apr	20-Oct
7-Apr	20-Oct
8-Apr	20-Oct
9-Apr	20-Oct
10-Apr	20-Oct
11-Apr	20-Oct
12-Apr	20-Oct
13-Apr	20-Oct
14-Apr	20-Oct
15-Apr	20-Oct
16-Apr	20-Oct
17-Apr	20-Oct
18-Apr	20-Oct
19-Apr	20-Oct
20-Apr	20-Oct
21-Apr	20-Oct
22-Apr	20-Oct
23-Apr	20-Oct
24-Apr	20-Oct
25-Apr	20-Oct
26-Apr	20-Oct
27-Apr	20-Oct
28-Apr	20-Oct
29-Apr	20-Oct
30-Apr	20-Oct
1-May	20-Oct
2-May	20-Oct
3-May	20-Oct
4-May	20-Oct
5-May	20-Oct
6-May	20-Oct
7-May	20-Oct
8-May	20-Oct
9-May	20-Oct
10-May	20-Oct

11-May	20-Oct
12-May	20-Oct
13-May	20-Oct
14-May	20-Oct
15-May	20-Oct
16-May	20-Oct
17-May	20-Oct
18-May	20-Oct
19-May	20-Oct
20-May	20-Oct
21-May	20-Oct
22-May	20-Oct
23-May	20-Oct
24-May	20-Oct
25-May	20-Oct
26-May	20-Oct
27-May	20-Oct
28-May	20-Oct
29-May	20-Oct
30-May	20-Oct
31-May	20-Oct
1-Jun	20-Oct
2-Jun	20-Oct
3-Jun	20-Oct
4-Jun	20-Oct
5-Jun	20-Oct
6-Jun	20-Oct
7-Jun	20-Oct
8-Jun	18-Nov
9-Jun	18-Nov
10-Jun	18-Nov
11-Jun	18-Nov
12-Jun	19-Nov
13-Jun	19-Nov
14-Jun	19-Nov
15-Jun	19-Nov
16-Jun	19-Nov
17-Jun	19-Nov
18-Jun	19-Nov
19-Jun	19-Nov
20-Jun	19-Nov
21-Jun	31-Dec
22-Jun	31-Dec

23-Jun	31-Dec
24-Jun	31-Dec
25-Jun	31-Dec
26-Jun	31-Dec
27-Jun	31-Dec
28-Jun	31-Dec
29-Jun	31-Dec
30-Jun	31-Dec
1-Jul	31-Dec
2-Jul	31-Dec
3-Jul	31-Dec
4-Jul	31-Dec
5-Jul	31-Dec
6-Jul	31-Dec
7-Jul	31-Dec
8-Jul	31-Dec
9-Jul	31-Dec
10-Jul	31-Dec
11-Jul	31-Dec
12-Jul	31-Dec
13-Jul	31-Dec
14-Jul	31-Dec
15-Jul	31-Dec
16-Jul	1-Jan
17-Jul	1-Jan
18-Jul	2-Jan
19-Jul	2-Jan
20-Jul	3-Jan
21-Jul	3-Jan
22-Jul	4-Jan
23-Jul	4-Jan
24-Jul	5-Jan
25-Jul	6-Jan
26-Jul	6-Jan
27-Jul	7-Jan
28-Jul	7-Jan
29-Jul	7-Jan
30-Jul	8-Jan
31-Jul	8-Jan
1-Aug	8-Jan
2-Aug	9-Jan
3-Aug	9-Jan
4-Aug	10-Jan

5-Aug	10-Jan
6-Aug	10-Jan
7-Aug	11-Jan
8-Aug	11-Jan
9-Aug	11-Jan
10-Aug	12-Jan
11-Aug	12-Jan
12-Aug	12-Jan
13-Aug	13-Jan
14-Aug	13-Jan
15-Aug	13-Jan
16-Aug	14-Jan
17-Aug	14-Jan
18-Aug	28-Feb
19-Aug	28-Feb
20-Aug	28-Feb
21-Aug	28-Feb
22-Aug	28-Feb
23-Aug	28-Feb
24-Aug	28-Feb
25-Aug	28-Feb
26-Aug	28-Feb
27-Aug	28-Feb
28-Aug	28-Feb
29-Aug	28-Feb
30-Aug	28-Feb
31-Aug	28-Feb
1-Sep	28-Feb
2-Sep	28-Feb
3-Sep	28-Feb
4-Sep	28-Feb
5-Sep	28-Feb
6-Sep	28-Feb
7-Sep	28-Feb
8-Sep	28-Feb
9-Sep	28-Feb
10-Sep	28-Feb
11-Sep	28-Feb
12-Sep	28-Feb
13-Sep	28-Feb
14-Sep	28-Feb
15-Sep	28-Feb
16-Sep	28-Feb

17-Sep	28-Feb
18-Sep	28-Feb
19-Sep	28-Feb
20-Sep	28-Feb
21-Sep	28-Feb
22-Sep	28-Feb
23-Sep	28-Feb
24-Sep	28-Feb
25-Sep	30-Apr
26-Sep	30-Apr
27-Sep	30-Apr
28-Sep	30-Apr
29-Sep	30-Apr
30-Sep	30-Apr
1-Oct	30-Apr
2-Oct	30-Apr
3-Oct	30-Apr
4-Oct	30-Apr
5-Oct	30-Apr
6-Oct	30-Apr
7-Oct	30-Apr
8-Oct	30-Apr
9-Oct	30-Apr
