# Supplementary material for: Optimal Annual COVID-19 Vaccine Boosting Dates Following Previous Booster Vaccination or Breakthrough Infection
Source: Clin Infect Dis. 2024 Nov 26;80(2):316–22. doi: 10.1093/cid/ciae559 (PMC11848277; doi:10.1093/cid/ciae559)
Supplement: ciae559_Supplementary_Data [file ciae559_supplementary_data.zip › TableS9_netherlands.pdf]

Supplementary Table S9	Netherlands
**Infection date**	**Following year optimal booster date**
22-Oct	22-Oct
23-Oct	22-Oct
24-Oct	22-Oct
25-Oct	23-Oct
26-Oct	23-Oct
27-Oct	23-Oct
28-Oct	24-Oct
29-Oct	24-Oct
30-Oct	24-Oct
31-Oct	25-Oct
1-Nov	25-Oct
2-Nov	25-Oct
3-Nov	26-Oct
4-Nov	26-Oct
5-Nov	26-Oct
6-Nov	27-Oct
7-Nov	27-Oct
8-Nov	27-Oct
9-Nov	28-Oct
10-Nov	28-Oct
11-Nov	28-Oct
12-Nov	29-Oct
13-Nov	29-Oct
14-Nov	29-Oct
15-Nov	30-Oct
16-Nov	30-Oct
17-Nov	30-Oct
18-Nov	31-Oct
19-Nov	1-Nov
20-Nov	1-Nov
21-Nov	2-Nov
22-Nov	2-Nov
23-Nov	3-Nov
24-Nov	3-Nov
25-Nov	4-Nov
26-Nov	4-Nov
27-Nov	5-Nov
28-Nov	5-Nov
29-Nov	6-Nov
30-Nov	6-Nov
1-Dec	6-Nov

2-Dec	7-Nov
3-Dec	7-Nov
4-Dec	7-Nov
5-Dec	8-Nov
6-Dec	8-Nov
7-Dec	8-Nov
8-Dec	9-Nov
9-Dec	9-Nov
10-Dec	9-Nov
11-Dec	10-Nov
12-Dec	10-Nov
13-Dec	10-Nov
14-Dec	11-Nov
15-Dec	11-Nov
16-Dec	11-Nov
17-Dec	12-Nov
18-Dec	12-Nov
19-Dec	12-Nov
20-Dec	13-Nov
21-Dec	13-Nov
22-Dec	13-Nov
23-Dec	14-Nov
24-Dec	14-Nov
25-Dec	14-Nov
26-Dec	15-Nov
27-Dec	15-Nov
28-Dec	15-Nov
29-Dec	16-Nov
30-Dec	16-Nov
31-Dec	16-Nov
1-Jan	16-Nov
2-Jan	16-Nov
3-Jan	16-Nov
4-Jan	16-Nov
5-Jan	17-Nov
6-Jan	17-Nov
7-Jan	17-Nov
8-Jan	17-Nov
9-Jan	17-Nov
10-Jan	17-Nov
11-Jan	17-Nov
12-Jan	18-Nov
13-Jan	18-Nov

14-Jan	18-Nov
15-Jan	18-Nov
16-Jan	18-Nov
17-Jan	18-Nov
18-Jan	18-Nov
19-Jan	18-Nov
20-Jan	19-Nov
21-Jan	19-Nov
22-Jan	19-Nov
23-Jan	19-Nov
24-Jan	19-Nov
25-Jan	19-Nov
26-Jan	19-Nov
27-Jan	19-Nov
28-Jan	20-Nov
29-Jan	20-Nov
30-Jan	20-Nov
31-Jan	20-Nov
1-Feb	20-Nov
2-Feb	20-Nov
3-Feb	20-Nov
4-Feb	21-Nov
5-Feb	21-Nov
6-Feb	21-Nov
7-Feb	21-Nov
8-Feb	21-Nov
9-Feb	21-Nov
10-Feb	21-Nov
11-Feb	21-Nov
12-Feb	22-Nov
13-Feb	22-Nov
14-Feb	22-Nov
15-Feb	22-Nov
16-Feb	22-Nov
17-Feb	22-Nov
18-Feb	22-Nov
19-Feb	22-Nov
20-Feb	22-Nov
21-Feb	23-Nov
22-Feb	23-Nov
23-Feb	23-Nov
24-Feb	23-Nov
25-Feb	23-Nov

26-Feb	23-Nov
27-Feb	24-Nov
28-Feb	24-Nov
1-Mar	24-Nov
2-Mar	24-Nov
3-Mar	24-Nov
4-Mar	24-Nov
5-Mar	24-Nov
6-Mar	25-Nov
7-Mar	25-Nov
8-Mar	25-Nov
9-Mar	25-Nov
10-Mar	8-Dec
11-Mar	8-Dec
12-Mar	9-Dec
13-Mar	9-Dec
14-Mar	10-Dec
15-Mar	10-Dec
16-Mar	11-Dec
17-Mar	11-Dec
18-Mar	12-Dec
19-Mar	12-Dec
20-Mar	13-Dec
21-Mar	13-Dec
22-Mar	14-Dec
23-Mar	14-Dec
24-Mar	15-Dec
25-Mar	15-Dec
26-Mar	16-Dec
27-Mar	16-Dec
28-Mar	17-Dec
29-Mar	17-Dec
30-Mar	18-Dec
31-Mar	18-Dec
1-Apr	19-Dec
2-Apr	19-Dec
3-Apr	20-Dec
4-Apr	20-Dec
5-Apr	21-Dec
6-Apr	21-Dec
7-Apr	21-Dec
8-Apr	22-Dec
9-Apr	22-Dec

10-Apr	23-Dec
11-Apr	23-Dec
12-Apr	24-Dec
13-Apr	24-Dec
14-Apr	25-Dec
15-Apr	25-Dec
16-Apr	26-Dec
17-Apr	26-Dec
18-Apr	27-Dec
19-Apr	27-Dec
20-Apr	28-Dec
21-Apr	28-Dec
22-Apr	29-Dec
23-Apr	29-Dec
24-Apr	30-Dec
25-Apr	30-Dec
26-Apr	31-Dec
27-Apr	1-Jan
28-Apr	1-Jan
29-Apr	2-Jan
30-Apr	2-Jan
1-May	3-Jan
2-May	3-Jan
3-May	4-Jan
4-May	4-Jan
5-May	5-Jan
6-May	5-Jan
7-May	6-Jan
8-May	7-Jan
9-May	7-Jan
10-May	7-Jan
11-May	8-Jan
12-May	8-Jan
13-May	9-Jan
14-May	9-Jan
15-May	9-Jan
16-May	10-Jan
17-May	10-Jan
18-May	11-Jan
19-May	11-Jan
20-May	11-Jan
21-May	12-Jan
22-May	12-Jan

23-May	13-Jan
24-May	13-Jan
25-May	14-Jan
26-May	14-Jan
27-May	14-Jan
28-May	15-Jan
29-May	15-Jan
30-May	16-Jan
31-May	16-Jan
1-Jun	16-Jan
2-Jun	17-Jan
3-Jun	17-Jan
4-Jun	17-Jan
5-Jun	17-Jan
6-Jun	17-Jan
7-Jun	18-Jan
8-Jun	18-Jan
9-Jun	18-Jan
10-Jun	18-Jan
11-Jun	18-Jan
12-Jun	19-Jan
13-Jun	19-Jan
14-Jun	19-Jan
15-Jun	19-Jan
16-Jun	19-Jan
17-Jun	20-Jan
18-Jun	20-Jan
19-Jun	20-Jan
20-Jun	20-Jan
21-Jun	20-Jan
22-Jun	21-Jan
23-Jun	21-Jan
24-Jun	21-Jan
25-Jun	21-Jan
26-Jun	21-Jan
27-Jun	21-Jan
28-Jun	22-Jan
29-Jun	22-Jan
30-Jun	22-Jan
1-Jul	22-Jan
2-Jul	22-Jan
3-Jul	23-Jan
4-Jul	23-Jan

5-Jul	23-Jan
6-Jul	23-Jan
7-Jul	23-Jan
8-Jul	23-Jan
9-Jul	24-Jan
10-Jul	24-Jan
11-Jul	24-Jan
12-Jul	24-Jan
13-Jul	25-Jan
14-Jul	25-Jan
15-Jul	25-Jan
16-Jul	25-Jan
17-Jul	25-Jan
18-Jul	8-Feb
19-Jul	9-Feb
20-Jul	9-Feb
21-Jul	10-Feb
22-Jul	10-Feb
23-Jul	11-Feb
24-Jul	11-Feb
25-Jul	12-Feb
26-Jul	12-Feb
27-Jul	13-Feb
28-Jul	14-Feb
29-Jul	15-Feb
30-Jul	15-Feb
31-Jul	16-Feb
1-Aug	17-Feb
2-Aug	18-Feb
3-Aug	19-Feb
4-Aug	19-Feb
5-Aug	20-Feb
6-Aug	21-Feb
7-Aug	22-Feb
8-Aug	22-Feb
9-Aug	23-Feb
10-Aug	24-Feb
11-Aug	25-Feb
12-Aug	25-Feb
13-Aug	26-Feb
14-Aug	27-Feb
15-Aug	27-Feb
16-Aug	28-Feb

17-Aug	28-Feb
18-Aug	28-Feb
19-Aug	28-Feb
20-Aug	28-Feb
21-Aug	28-Feb
22-Aug	28-Feb
23-Aug	28-Feb
24-Aug	28-Feb
25-Aug	1-Mar
26-Aug	1-Mar
27-Aug	2-Mar
28-Aug	2-Mar
29-Aug	3-Mar
30-Aug	3-Mar
31-Aug	4-Mar
1-Sep	4-Mar
2-Sep	5-Mar
3-Sep	5-Mar
4-Sep	6-Mar
5-Sep	6-Mar
6-Sep	7-Mar
7-Sep	8-Mar
8-Sep	9-Mar
9-Sep	9-Mar
10-Sep	10-Mar
11-Sep	11-Mar
12-Sep	11-Mar
13-Sep	12-Mar
14-Sep	13-Mar
15-Sep	14-Mar
16-Sep	31-Mar
17-Sep	31-Mar
18-Sep	31-Mar
19-Sep	31-Mar
20-Sep	31-Mar
21-Sep	31-Mar
22-Sep	31-Mar
23-Sep	31-Mar
24-Sep	31-Mar
25-Sep	31-Mar
26-Sep	31-Mar
27-Sep	31-Mar
28-Sep	31-Mar

29-Sep	31-Mar
30-Sep	31-Mar
1-Oct	31-Mar
2-Oct	31-Mar
3-Oct	31-Mar
4-Oct	31-Mar
5-Oct	31-Mar
6-Oct	31-Mar
7-Oct	31-Mar
8-Oct	31-Mar
9-Oct	31-Mar
10-Oct	31-Mar
11-Oct	31-Mar
12-Oct	31-Mar
13-Oct	31-Mar
14-Oct	31-Mar
15-Oct	31-Mar
16-Oct	31-Mar
17-Oct	31-Mar
18-Oct	1-Apr
19-Oct	1-Apr
20-Oct	2-Apr
21-Oct	3-Apr
